# Morphology and multilocus phylogeny illuminate novel taxa in *Capnodiaceae* (*Ascomycota*, *Dothideomycetes*, *Capnodiales*)

**DOI:** 10.3897/imafungus.17.188051

**Published:** 2026-05-29

**Authors:** Jipeng Sun, Yuwei Liu, Tao Chen, Entaj Tarafder, Sinang Hongsanan, Shengxi Chen, Chada Norphanphoun, Xiaolu Deng, Yuzhe Feng, Tian Yang, Samantha C. Karunarathna, Xiangyu Zeng

**Affiliations:** 1 Department of Plant Pathology, College of Agriculture, Guizhou University, Guiyang 550025, China College of Life Science and Oceanography, Shenzhen University Shenzhen China; 2 Guizhou Key Laboratory of Agricultural Microbiology, Guiyang 550025, China College of Biology and Food Engineering, Qujing Normal University Qujing China; 3 Institute of Edible Mushrooms, Guizhou University, Guiyang 550025, China Department of Plant Pathology, College of Agriculture, Guizhou University Guiyang China; 4 School of Public Health, Guizhou Medical University, Guiyang 561113, China Institute of Edible Mushrooms, Guizhou University Guiyang China; 5 Center for Yunnan Plateau Biological Resources Protection and Utilization & Yunnan International Joint Laboratory of Fungal Sustainable Utilization in South and Southeast Asia, College of Biology and Food Engineering, Qujing Normal University, Qujing 655099, China School of Public Health, Guizhou Medical University Guiyang China; 6 Shenzhen Key Laboratory of Microbial Genetic Engineering, College of Life Science and Oceanography, Shenzhen University, Shenzhen 518060, China Guizhou Key Laboratory of Agricultural Microbiology Guiyang China

**Keywords:** Fungal systematics, molecular phylogenetics, morphological variation, sooty mold fungi, species delimitation

## Abstract

*Capnodiaceae* is a major family of sooty mold fungi, but its species diversity, host associations, and geographic distribution remain insufficiently documented in China. Many taxa in this family have been described based on limited morphological information or incomplete molecular datasets, resulting in unresolved generic and species boundaries. In this study, newly collected *Capnodiaceae* material from China was examined using morphological observations, culture characteristics, host and geographic information, and four-locus phylogenetic analyses based on ITS, LSU, *tef*1-α, and *rpb*2 sequence data. A total of 37 newly collected strains associated with 25 host plant taxa belonging to 23 genera and 19 families were included. Phylogenetic analyses resolved several well-supported lineages within *Capnodiaceae* and supported the recognition of one new genus, *Hyphopolychaeton*, and eight new species in *Conidiocarpus*, *Leptoxyphium*, *Polychaeton*, and *Hyphopolychaeton*. In addition, 11 new host records and three new records for China are documented. Host and geographic information showed that several taxa, including *Conidiocarpus cinnamomi* and *C. caucasicus*, occur on multiple host plants, indicating that host association alone should not be used as a primary criterion for species delimitation. Morphological comparisons showed that the position and structure of the conidiogenous region within the conidioma are informative for generic delimitation when congruent with multilocus phylogenetic evidence. In contrast, minor quantitative differences in conidiomatal or conidial dimensions may represent intraspecific variation when not supported by clear molecular divergence. An expanded *Leptoxyphium* phylogeny including all available unnamed strains further supports a conservative interpretation of species boundaries in this genus. Overall, this study provides new morphological, ecological, host-associated, geographic, and multilocus molecular data for Chinese *Capnodiaceae* and strengthens the reference framework for species recognition and generic delimitation in sooty mold fungi.

## Introduction

Sooty mold fungi are ecologically important epiphytic fungi that commonly occur on the surfaces of leaves, branches, stems, flowers, and fruits, where they form dark, soot-like mycelial layers. These fungi usually develop on honeydew excreted by sap-sucking insects, such as scale insects, aphids, and whiteflies, and may affect plant appearance, photosynthesis, and growth by covering the host surface (Hughes 1976; Chomnunti et al. 2014; Ariyawansa et al. 2015; Hongsanan et al. 2015). Although sooty molds represent an ecological group rather than a single taxonomic lineage, several families within *Ascomycota* include sooty mold taxa. Among them, *Capnodiaceae* is one of the most representative families and includes many taxa associated with subtropical and tropical plant surfaces.

*Capnodiaceae* was introduced by Höhnel (1910), with *Capnodium* designated as the type genus (Hyde et al. 2013; Wijayawardene et al. 2014; Theissen 1915; Yang et al. 2025). Prior to the present study, *Capnodiaceae* comprised 11 accepted genera, namely *Capnodium*, *Chaetocapnodium*, *Conidiocarpus*, *Heteroconium*, *Hyphocapnodia*, *Kosmimatomyces*, *Leptoxyphium*, *Limaciniaseta*, *Phragmocapnias*, *Polychaeton*, and *Scoriadopsis* (Chomnunti et al. 2011; Abdollahzadeh et al. 2020; Lu et al. 2022; Wijayawardene et al. 2022; Yang et al. 2025). However, molecular data remain unavailable or limited for several genera, including *Kosmimatomyces*, *Limaciniaseta*, and *Scoriadopsis*, resulting in uncertain phylogenetic placements and poorly resolved generic boundaries. Although a short SSU sequence has been reported for *Limaciniaseta californica*, this single marker is insufficient to reliably infer its phylogenetic position (Reynolds 1998). More generally, previous studies have shown that reliance solely on ITS and LSU sequences is often inadequate for resolving closely related taxa in *Capnodiaceae* (Abdollahzadeh et al. 2020; Czachura et al. 2025). Therefore, phylogenetic studies incorporating additional gene regions are necessary to improve generic delimitation and clarify evolutionary relationships within the family.

Members of *Capnodiaceae* are characterized by superficial, septate, dark brown hyphae that form a thin, often sooty mycelial layer on plant surfaces. Both sexual and asexual morphs may occur on the same host or on different hosts (Jouraeva et al. 2006; Chomnunti et al. 2011; Hyde et al. 2013). Sexual morphs are typically formed within a mycelial matrix and consist of subglobose to globose ascomata, which may be setose or asetose and possess a central ostiole. The peridium is brown, thin-walled, and composed of *textura angularis*, with pseudoparaphyses absent. Asci are 8-spored, bitunicate, saccate, short-pedicellate, and typically lack an ocular chamber (Chomnunti et al. 2011). Ascospores are bi- to triseriate, hyaline to brown, and multiseptate or muriform. Asexual morphs may be coelomycetous or hyphomycetous (Chomnunti et al. 2011; Abdollahzadeh et al. 2020). Conidiomata are synnematous or pycnidial, globose to pyriform or elongate, with or without necks or swollen regions. Conidia are usually hyaline, aseptate, and ellipsoid.

Morphological comparisons in *Capnodiaceae* are often constrained by the morphs available for each taxon. Many species are known mainly from asexual morphs, whereas some taxa, such as *Phragmocapnias plumeriae* (= *Conidiocarpus plumeriae*) and *P. betle* (= *C. betle*), have been compared primarily using sexual characters, including asci and ascospore septation, because their asexual morphs are unknown or insufficiently documented (Hongsanan et al. 2015; Abdollahzadeh et al. 2020). This heterogeneity in available morphological stages makes direct character-by-character comparisons among taxa difficult and highlights the need for integrative evidence combining morphology and multilocus phylogeny.

Recent studies have highlighted the taxonomic complexity and unresolved generic boundaries within *Capnodiaceae*, underscoring the need for comprehensive taxonomic revision (Abdollahzadeh et al. 2020). One major challenge is the frequent co-occurrence of multiple sooty mold taxa on the same host surface, which often results in mixed collections and complicates species delimitation and morphological description (Khodaparast et al. 2020). These difficulties are further exacerbated by the high morphological diversity and complex life cycles observed across the family. In addition, the connections between sexual and asexual morphs remain poorly understood for many taxa (Chomnunti et al. 2014). Consequently, species delimitation in *Capnodiaceae* requires careful comparison of morphology, molecular phylogeny, host association, and geographic distribution.

Despite its exceptional biodiversity, China remains under-sampled for *Capnodiaceae*. To our knowledge, the currently confirmed *Capnodiaceae* taxa from China with clear taxonomic placement and available molecular evidence include five newly described species and one newly recorded species. These include *Conidiocarpus fici-septicae* from fallen leaves of *Ficus septica* (Tennakoon et al. 2021), *Hyphocapnodia sichuanensis* from Sichuan Province, representing the only known species of *Hyphocapnodia* (Lu et al. 2022), *Conidiocarpus cinnamomi* from leaves of *Cinnamomum japonicum* (Yang et al. 2025), *Conidiocarpus caucasicus* from withered leaves of *Cryptomeria japonica* in southwestern China (Tian et al. 2024), and the recently described Chinese taxa *Conidiocarpus nanshanense* from leaves of *Schefflera macrostachya* in Guizhou Province and *Polychaeton cengongense* from leaves of *Dalbergia assamica* in Guangdong Province (Sun et al. 2026). Collectively, these limited records indicate that *Capnodiaceae* remains markedly underrepresented in China and suggest that the diversity of sooty mold fungi in this region is still poorly documented.

In the present study, *Capnodiaceae* taxa collected from several provinces in China were investigated using a combined morphological and four-locus phylogenetic framework. Morphological observations, culture characteristics, host information, and four-locus phylogenetic analyses based on ITS, LSU, *tef*1-α, and *rpb*2 are combined to evaluate generic and species boundaries. The aims of this study are to describe novel taxa, document new host and geographic records, clarify the placement of selected poorly known lineages, and assess the diagnostic value of morphological characters within a multilocus phylogenetic framework. This work provides new reference data for *Capnodiaceae* and contributes to a more robust classification of sooty mold fungi.

## Materials and methods

### Sample collection, isolation, and morphological observations

Sooty mold-infected leaves were collected from various host plants across Guizhou, Zhejiang, Guangdong, and Fujian Provinces, China, throughout all seasons from 2022 to 2025, with sampling predominantly concentrated in Guizhou Province and conducted in diverse habitats, including public parks, natural scenic areas, botanical gardens, and primeval forests at each locality. The samples were placed in paper envelopes, transported to the laboratory, and stored at 4 °C. Macromorphological characteristics, including colony characteristics (size, shape, elevation, margin, texture, and color) and the distribution of conidiomata, were observed and documented using a stereomicroscope (Keyence VHX-7000 Digital Microscope, Japan). For microscopic analysis, samples were prepared on slides with sterile water and examined under a compound optical microscope (Zeiss Axioscope 5, Germany) equipped with an AxioCam 208 color camera and a phase-contrast optical system. The shape, pigmentation, and dimensions of hyphae, conidiomata, ostioles, the position of the swollen region of the conidioma, and conidia were photographed and recorded. All images for illustration and dimensional measurements were processed using Adobe Photoshop (2023, 24.0.0.59). The holotype was deposited at the Herbarium of IFRD (International Fungal Research & Development Centre; Institute of Highland Forest Science, Chinese Academy of Forestry, Kunming, China). The ex-type living culture was deposited at the Culture Collection of the Herbarium of IFRD (IFRDCC). The newly described species were registered in Index Fungorum, and the corresponding accession numbers were obtained (Index Fungorum 2026).

### Isolation and cultural characteristics

Single conidia were aseptically isolated from fresh conidiomata or conidial masses under a stereomicroscope using a sterile needle and placed onto potato dextrose agar (PDA). Germinating single-conidium colonies were transferred to fresh PDA plates to establish pure cultures. Cultures were incubated at 26 °C, and colony characteristics were recorded after 14 d. Colony diameter, color, texture, margin, and pigmentation on both the surface and reverse sides were noted. Micromorphological characters produced in culture were observed under a compound microscope, and measurements were made whenever possible.

### DNA extraction, PCR amplification, and sequencing

DNA extraction was performed with fresh fungal mycelia cultured on PDA at 26 °C in darkness for 2 weeks using the BEIWO Fungal DNA Extraction Kit (Hangzhou Beiwo Medical Technology Co., Ltd., catalog No. BW-GD2416), following the manufacturer’s protocol. Genomic DNA of the host plant was extracted using the Plant Genomic DNA Extraction Kit from Beijing Solarbio Science & Technology Co., Ltd. For fungal gene amplification, four molecular markers were targeted: the internal transcribed spacer (ITS) region, including 5.8S rDNA, using primers ITS1/ITS4 (White et al. 1990); the large subunit nuclear ribosomal DNA region (LSU), using primers LR0R (Rehner and Samuels 1994) and LR5 (Vilgalys and Hester 1990); the translation elongation factor 1-α gene (*tef*1-α), using primers EF1-983F/EF1-2218R (Reynolds and Gilbert 2005); and the RNA polymerase II second largest subunit gene (*rpb*2), using primers RPB2-5F/RPB2-7cR (Liu et al. 1999). For the host plant, the partial *rbcL* gene was amplified using the primer pair SI_Forward and SI_Reverse, which was developed by Kress et al. (2009). The PCR reaction mixture had a total volume of 20 μL, consisting of 17 μL GoldenStar T6 Super PCR Mix Ver. 2 (1.1×), 1 μL DNA template, 1 μL forward primer, and 1 μL reverse primer. PCR cycling parameters were optimized for each molecular marker, with the key annealing temperatures specified as follows: ITS and LSU were amplified with an annealing temperature of 52 °C for 45 s (35 cycles), *tef*1-α at 55 °C for 45 s (35 cycles), and *rpb*2 with an annealing temperature of 52 °C for 2 min (35 cycles). Other standard PCR conditions included initial denaturation, denaturation, extension, and final hold steps, as described in the original protocols (Vilgalys and Hester 1990; White et al. 1990; Reynolds and Gilbert 2005; Kress et al. 2009). Detailed procedures for the PCR assays used in the present study are listed in Table 1. PCR products were analyzed using 1% agarose gel, which was prepared and, after complete solidification, placed in an electrophoresis tank containing buffer. DNA samples were loaded into the wells, and electrophoresis was performed at 100 V for 30 min. The gel was visualized using a gel imaging system. PCR products were considered qualified if clear, bright bands were observed at the expected positions corresponding to the target gene fragments. Qualified PCR products were sent to Tsingke Biotechnology Co., Ltd. (Beijing) for sequencing. The resulting valid sequences were deposited in GenBank (Table 2).

**Table 1.** PCR conditions and the primers used in this study.

Gene	Primers	Sequence (5'–3')	PCR cycles	References
ITS	ITS1	TCCGTAGGTGAACCTGCGG	(95 °C: 30 s, 52 °C: 45 s, 72 °C: 60 s) × 35 cycles	(White et al. 1990)
	ITS4	TCCTCCGCTTATTGATATGC		
LSU	LR0R	ACCCGCTGAACTTAAGC	(95 °C: 30 s, 52 °C: 45 s, 72 °C: 60 s) × 35 cycles	(Vilgalys and Hester 1990; Rehner and Samuels 1994)
	LR5	ATCCTGAGGGAAACTTC		
*tef1-α*	EF1-983F	GCYCCYGGHCAYCGTGAYTTYAT	(95 °C: 30 s, 55 °C: 45 s, 72 °C: 60 s) × 35 cycles	(Reynolds and Gilbert 2005)
	EF1-2218R	ATGACACCRACRGCRACRGTYTG		
*rpb2*	RPB2-5F	GAYGAYMGWGATCAYTTYGG	(95 °C: 1 min, 52 °C: 2 min, 72 °C: 90 s) × 35 cycles	(Liu et al. 1999; Reeb et al. 2004; O’Donnell et al. 2007; Yang et al. 2025)
	RPB2-7cR	CCCATRGCTTGYTTRCCCAT		
*rbcL*	SI_Forward	ATGTCACCACAAACAGAGACTAAAGC	(94 °C: 30 s, 55 °C: 30 s, 72 °C: 60 s) × 35 cycles	(Kress et al. 2009)
	SI_Reverse	GTAAAATCAAGTCCACCRCG		

**Table 2.** Taxa used in the phylogenetic analysis of *Capnodiaceae* and their corresponding GenBank accession numbers.

Species	Strain	GenBank accession number				Reference
		ITS	LSU	*tef1-*α	*rpb*2	
*Capnodium aciculiforme*	CBS 892.73	-	-	GU349045	-	Schoch et al. (2009)
*Capnodium alfenasii* ^T^	CBS 146151	MN749233	MN749165	MN829346	MN829260	Bezerra et al. (2017); Abdollahzadeh et al. (2020)
*Capnodium blackwelliae* ^T^	CBS 133588	MN749235	-	-	-	Abdollahzadeh et al. (2020)
*Capnodium coartatum*	CPC 17779	MN749236	MN749167	MN829348	MN829262	Hyde et al. (2016)
*Capnodium coartatum*	SDBR-CMU477	OR458575	OR458609	OR523660	-	Thungdee et al. (2023)
*Capnodium coffeae* ^T^	CBS 147.52	DQ491515	GU214400	DQ471089	KT216519	Spatafora et al. (2006)
*Capnodium coffeicola* ^T^	MFLUCC 15-0206	KU358921	KU358920	-	-	Hongsanan et al. (2015)
*Capnodium gamsii*	CBS 146153	MN749238	MN749168	MN829349	MN829263	Abdollahzadeh et al. (2020)
*Capnodium gardeniarum*	CPC 14327	-	GU301807	GU349054	GU371743	Schoch et al. (2009); Li et al. (2020)
*Capnodium neocoffeicola*	IFRDCC 19-1201	PX352582	PX352578	PX354087	PX942497	This study
*Capnodium neocoffeicola* ^T^	CBS 139614	MN749242	MN749172	MN829353	MN829267	Abdollahzadeh et al. (2020)
*Capnodium paracoartatum*	MFLUCC 14-0282	NR_169716	NG_073832	-	-	Li et al. (2020)
*Capnodium paracoffeicola* ^T^	CBS 139616	MN749244	MN749174	MN829355	MN829269	Abdollahzadeh et al. (2020)
*Chaetocapnodium thailandense* ^T^	CBS 139619	MN749254	MN749183	MN829366	MN829281	Abdollahzadeh et al. (2020)
*Chaetocapnodium insulare* ^T^	CBS 146159	MN749248	NG_068681	-	-	Abdollahzadeh et al. (2020)
*Chaetocapnodium magnum* ^T^	CBS 153154	PV583761	PV583758	PV591868	PV591871	Czachura et al. (2025)
*Chaetocapnodium polonicum* ^T^	CBS 153156	PV583763	PV583760	PV591870	PV591873	Czachura et al. (2025)
*Chaetocapnodium siamensis* ^T^	MFLUCC 13-0778	-	KP744479	-	-	Abdollahzadeh et al. (2020)
*Chaetocapnodium tanzanicum* ^T^	CBS 145.79	MN749253	MN749182	MN829365	MN829280	Abdollahzadeh et al. (2020)
*Conidiocarpus bijieense*	IFRDCC 19-1186	PX308074	PX308120	PX317153	PX942492	This study
*Conidiocarpus bijieense* ^T^	IFRDCC 19-1189	PX352580	PX352576	PX354085	-	This study
*Conidiocarpus caucasicus*	IFRDCC 19-1159	PX929793	PX929821	PX934663	PX934691	This study
*Conidiocarpus caucasicus*	IFRDCC 25-0006	PX929794	PX929822	PX934664	PX934692	This study
*Conidiocarpus caucasicus*	IFRDCC 19-1195	PX929795	PX929823	PX934665	PX934693	This study
*Conidiocarpus caucasicus*	UESTCC 23.0246	OR887382	OR887093	PP076823	PP076815	Tian et al. (2024)
*Conidiocarpus caucasicus*	IFRDCC 19-1192	PX307107	PX308113	PX317146	PX942493	This study
*Conidiocarpus cinnamomi*	IFRDCC 19-1150	PX929787	PX929815	PX934657	PX934685	This study
*Conidiocarpus cinnamomi*	IFRDCC 19-1174	PX929788	PX929816	PX934658	PX934686	This study
*Conidiocarpus cinnamomi*	IFRDCC 19-1168	PX929789	PX929817	PX934659	PX934687	This study
*Conidiocarpus cinnamomi*	IFRDCC 25-0005	PX929790	PX929818	PX934660	PX934688	This study
*Conidiocarpus cinnamomi*	IFRDCC 19-1165	PX929791	PX929819	PX934661	PX934689	This study
*Conidiocarpus cinnamomi*	IFRDCC 19-1171	PX929792	PX929820	PX934662	PX934690	This study
*Conidiocarpus cinnamomi* ^T^	SICAU 23-0100	PP736390	PP732745	PP779591	PP782090	Yang et al. (2025)
*Conidiocarpus fici-septicae* ^T^	MFLUCC 19-0072	MW063143	MW063206	-	-	Tennakoon et al. (2021)
*Conidiocarpus guilanensis*	IFRDCC 19-1213	PX352581	PX352577	PX354086	-	This study
*Conidiocarpus guilanensis*	IFRDCC 25-0007	PX929799	PX929827	PX934669	PX934696	This study
*Conidiocarpus guilanensis* ^T^	IRAN 2474C	MG906804	-	-	-	Khodaparast et al. (2020)
*Conidiocarpus luodianense*	IFRDCC 19-1153	PX929798	PX929826	PX934668	PX934695	This study
*Conidiocarpus luodianense*	IFRDCC 19-1177	PX929797	PX929825	PX934667	PX934694	This study
*Conidiocarpus luodianense* ^T^	IFRDCC 19-1183	PX308072	PX308118	PX317151	PX942495	This study
*Conidiocarpus nanshanense* ^T^	IFRDCC 25-0001	PX668540	PX668542	PX682093	PX687868	Sun et al. (2026)
*Conidiocarpus tainingense* ^T^	IFRDCC 19-1198	PX307238	PX308114	PX317147	PX942494	This study
*Conidiocarpus* sp.	CBS 139818	MN749261	MN749190	MN829373	MN829288	Abdollahzadeh et al. (2020)
*Conidiocarpus* sp.	CBS 139821	MN749260	MN749189	MN829372	MN829287	Abdollahzadeh et al. (2020)
*Conidiocarpus* sp.	CBS 139819	MN749263	MN749192	MN829375	MN829290	Abdollahzadeh et al. (2020)
*Conidiocarpus* sp.	CBS 139820	MN749255	MN749184	MN829367	MN829282	Abdollahzadeh et al. (2020)
*Conidiocarpus* sp.	CPC 20463	MN749258	MN749187	MN829370	MN829285	Abdollahzadeh et al. (2020)
*Conidiocarpus* sp.	CPC 20465	MN749262	MN749191	MN829374	MN829289	Abdollahzadeh et al. (2020)
*Conidiocarpus* sp.	CPC 20472	MN749259	MN749188	MN829371	MN829286	Abdollahzadeh et al. (2020)
*Conidiocarpus* sp.	CPC 17778	MN749256	MN749185	MN829368	MN829283	Abdollahzadeh et al. (2020)
*Conidiocarpus* sp.	CPC 21380	MN749257	MN749186	MN829369	MN829284	Abdollahzadeh et al. (2020)
*Heteroconium citharexyli* ^T^	CPC 13957	HM628776	HM628775	-	-	Cheewangkoon et al. (2012)
*Hyphocapnodia sichuanensis* ^T^	CGMCC 3.23573	ON603981	ON603986	ON646697	ON646698	Lu et al. (2022)
*Hyphopolychaeton cengongense*	IFRDCC 25-0017	PX929809	PX929837	PX934679	PX934706	This study
*Hyphopolychaeton cengongense*	IFRDCC 25-0018	PX929810	PX929838	PX934680	PX934707	This study
*Hyphopolychaeton cengongense*	IFRDCC 25-0019	PX929811	PX929839	PX934681	PX934708	This study
*Hyphopolychaeton cengongense* ^T^	IFRDCC 25-0020	PX929812	PX929840	PX934682	PX934709	This study
*Hyphopolychaeton duyunense*	IFRDCC 25-0008	PX929800	PX929828	PX934670	PX934697	This study
*Hyphopolychaeton duyunense*	IFRDCC 25-0009	PX929801	PX929829	PX934671	PX934698	This study
*Hyphopolychaeton duyunense*	IFRDCC 25-0010	PX929802	PX929830	PX934672	PX934699	This study
*Hyphopolychaeton duyunense*	IFRDCC 25-0012	PX929804	PX929832	PX934674	PX934701	This study
*Hyphopolychaeton duyunense*	IFRDCC 25-0013	PX929805	PX929833	PX934675	PX934702	This study
*Hyphopolychaeton duyunense*	IFRDCC 25-0014	PX929806	PX929834	PX934676	PX934703	This study
*Hyphopolychaeton duyunense*	IFRDCC 25-0015	PX929807	PX929835	PX934677	PX934704	This study
*Hyphopolychaeton duyunense*	IFRDCC 25-0016	PX929808	PX929836	PX934678	PX934705	This study
*Hyphopolychaeton maolanense* ^T^	IFRDCC 25-0021	PX929813	PX929841	PX934683	PX934710	This study
*Hyphopolychaeton duyunense* ^T^	IFRDCC 25-0011	PX929803	PX929831	PX934673	PX934700	This study
*Leptoxyphium madagascariense* ^T^	CBS 124766	MH863407	MH874923	MN829380	MN829296	Abdollahzadeh et al. (2020)
*Leptoxyphium chishuiense*	IFRDCC 19-1225	PX929814	PX929842	PX934684	PX934711	This study
*Leptoxyphium chishuiense* ^T^	IFRDCC 19-1222	PX307239	PX308115	PX317148	PX942499	This study
*Leptoxyphium citri*	CBS 146162	MN749267	MN749195	MN829378	-	Abdollahzadeh et al. (2020)
*Leptoxyphium citri* ^T^	CBS 451.66	MN749266	KF902094	GU349039	GU371727	Abdollahzadeh et al. (2020)
*Leptoxyphium fumago*	CBS 123.26	MH854862	GU214430	GU349051	GU371741	Abdollahzadeh et al. (2020)
*Leptoxyphium fumago*	IFRDCC 19-1219	PX352579	PX352575	PX354084	PX942498	This study
*Leptoxyphium kurandae* ^T^	CBS 129530	JF951150	JF951170	MN829379	MN829295	Crous et al. (2011)
*Leptoxyphium* sp.	CBS 139618	MN749276	MN749204	MN829389	MN829305	Abdollahzadeh et al. (2020)
*Leptoxyphium* sp.	CPC 17767	MN749275	MN749203	MN829388	MN829304	Abdollahzadeh et al. (2020)
*Leptoxyphium* sp.	CBS 135836	MN749278	MN749206	MN829391	MN829307	Abdollahzadeh et al. (2020)
*Leptoxyphium* sp.	CBS 139620	MN749279	MN749207	MN829392	MN829308	Abdollahzadeh et al. (2020)
*Phaeoxyphiella australiana* ^T^	CBS 146169	MN749292	MN749220	MN829406	MN829322	Abdollahzadeh et al. (2020)
*Phaeoxyphiella phylicae* ^T^	CBS 146170	MN749291	MN749219	MN829405	MN829321	Abdollahzadeh et al. (2020)
*Phragmocapnias betle*	CPC 17762	MN749293	MN749221	MN829407	MN829323	Abdollahzadeh et al. (2020)
*Phragmocapnias betle*	MFLUCC 10-0050	-	JN832605	-	-	Bose et al. (2014)
*Phragmocapnias betle* ^ET^	MFLUCC 10-0053	KU358922	JN832606	-	-	(Bose et al. 2014)
*Phragmocapnias plumeriae* ^T^	MFLUCC 15-0205	KU358919	KU358918	-	-	Hongsanan et al. (2015)
*Polychaeton cengongense*	IFRDCC 25-0004	PX953032	PX953035	PX961901	PX961902	Sun et al. (2026)
*Polychaeton cengongense* ^T^	IFRDCC 25-0002	PX668539	PX668541	PX682092	PX682094	Sun et al. (2026)
*Polychaeton citri*	CBS 116435	GU214649	GU214469	MN829394	MN829310	Crous et al. (2009)
*Polychaeton citruscola* ^T^	IFRDCC 19-1216	PX308073	PX308119	PX317152	-	This study
*Scolecoxyphium blechnicola* ^T^	CBS 146175	MN749297	MN749225	MN829413	MN829329	Abdollahzadeh et al. (2020)
*Scolecoxyphium blechni* ^T^	CBS 146174	MN749296	MN749224	MN829412	MN829328	Abdollahzadeh et al. (2020)
*Scolecoxyphium leucadendri* ^T^	CBS 146176	MN749298	MN749226	MN829414	MN829330	Abdollahzadeh et al. (2020)
*Scolecoxyphium phylicae* ^T^	CBS 146177	MN749299	MN749227	MN829415	MN829331	Abdollahzadeh et al. (2020)

Sequences and details of strains obtained in this study are shown in bold. Superscripts T and ET indicate ex-type and ex-epitype strains, respectively. - indicates unavailable sequence data.

### Phylogenetic analyses

Gene sequencing results obtained from the biotechnology company were analyzed by examining DNA sequence peaks using BioEdit version 7.2.5 (Hall 1999). Phylogenetic analyses were performed using One-stop phylogenetic tools (OFPT) (Zeng et al. 2023), following recent applications of this workflow in fungal molecular identification and phylogenetic analyses (Xie et al. 2024). Reference sequences were downloaded from the NCBI database (https://www.ncbi.nlm.nih.gov/) and combined with newly generated sequences. Datasets for each gene region were aligned independently using MAFFT (https://mafft.cbrc.jp/alignment/server/) (Katoh and Standley 2013), and the alignments were trimmed using TrimAl with the “gappyout” method (Capella-Gutiérrez et al. 2009). The aligned loci were then concatenated for multilocus phylogenetic analyses. Maximum likelihood (ML) analyses were performed using the IQ-TREE module implemented in OFPT (Zeng et al. 2023), following the default workflow of the software. The best-fit nucleotide substitution model for each partition was selected using the ModelFinder function under the Bayesian information criterion (BIC), and branch support for the ML tree was assessed using 1000 ultrafast bootstrap replicates. Bayesian inference (BI) analyses were conducted in MrBayes v. 3.2.6 (Ronquist et al. 2012) using a Markov chain Monte Carlo (MCMC) approach with two independent runs of four Markov chains each. The analysis was run for 50 million generations, sampling every 100 generations, and was terminated when the average standard deviation of split frequencies between runs dropped below 0.01. The first 25% of trees were discarded as burn-in, and posterior probabilities (PP) were calculated for each clade. The resulting tree files were visualized using FigTree v. 1.4.3 and further edited in Adobe Illustrator CC 2024.

## Results

### Phylogenetic analyses

The alignment included 93 strains and contained a total of 3002 characters, including gaps, with 1–389, 390–1,167, 1,168–2,199, and 2,200–3,002 corresponding to ITS, LSU, *rpb*2, and *tef*1-α, respectively. The optimal nucleotide substitution models were TNe+G4, TNe+R2, TIM2e+I+G4, and TIM3+F+I+G4, respectively. The phylogenetic analysis of the concatenated dataset produced a best-scoring tree with a final maximum likelihood optimization value of –17602.223.

**Figure 1.**
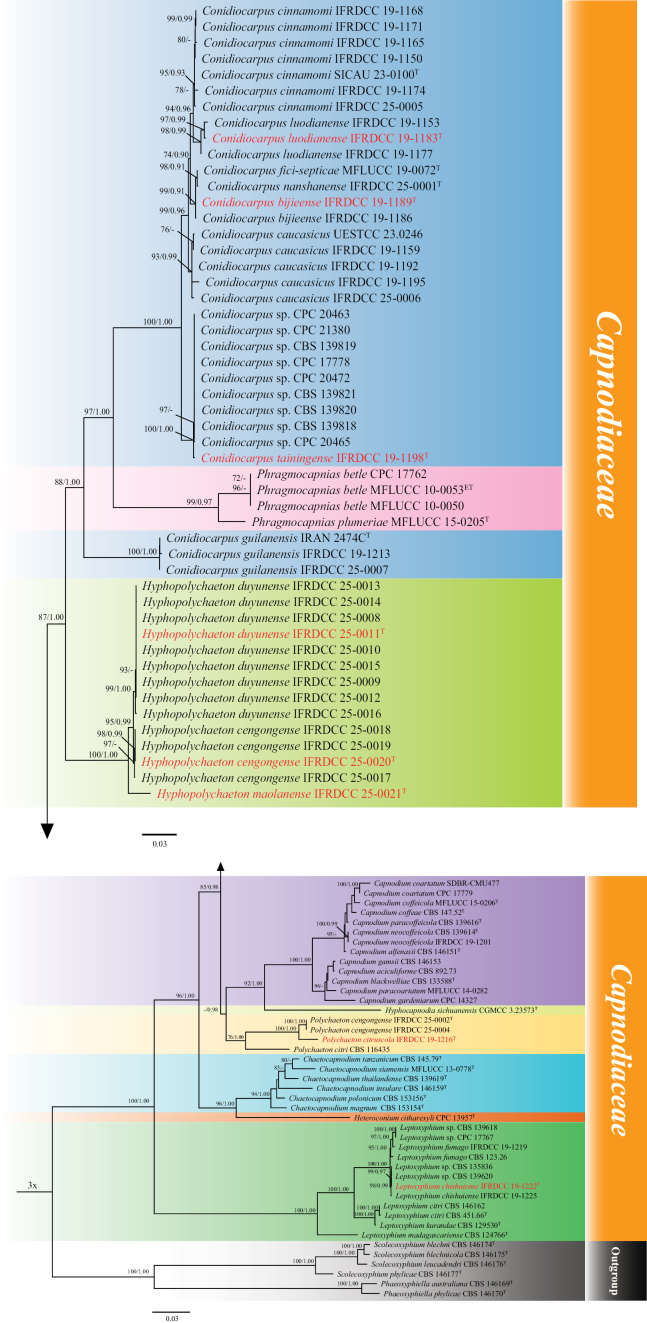
Phylogenetic tree inferred from maximum likelihood (ML) analysis based on the concatenated LSU, ITS, *tef*1-α, and *rpb*2 dataset. The tree was rooted with representatives of *Readerielliopsidaceae* as the outgroup (Abdollahzadeh et al. 2020). Bootstrap support (BS ≥ 70%) and Bayesian posterior probabilities (PP ≥ 0.90) are shown at the nodes. Ex-type and ex-epitype strains are indicated by superscripts T and ET, respectively. Type strains of taxa newly introduced in this study are shown in red.

The topology of the BI tree was congruent with that of the ML tree; therefore, only the ML tree is presented (Fig. 1). *Capnodiaceae* was resolved into nine generic lineages represented by *Conidiocarpus* (32 representative sequences), *Phragmocapnias* (4), *Hyphopolychaeton* (14), *Capnodium* (13), *Hyphocapnodia* (1), *Polychaeton* (4), *Chaetocapnodium* (6), *Heteroconium* (1), and *Leptoxyphium* (12). Multi-sample genera were generally recovered as distinct lineages, whereas *Hyphocapnodia* and *Heteroconium* were each represented by a single sequence. In this study, three new species are recognized in *Conidiocarpus*, namely *C. luodianense*, *C. bijieense*, and *C. tainingense*. Additional Chinese collections assigned to *C. cinnamomi*, *C. caucasicus*, and *C. guilanensis* provide new host and geographic records for the genus. The newly introduced genus *Hyphopolychaeton* formed an independent lineage and includes three new species, *Hy. duyunense*, *Hy. cengongense*, and *Hy. maolanense*. In addition, one new species is described in *Polychaeton* (*P. citruscola*) and one in *Leptoxyphium* (*L. chishuiense*). *Capnodium neocoffeicola* and *L. fumago* are represented by newly collected Chinese material, providing additional host and geographic information for these taxa.

### Host and geographic associations of the examined taxa

A total of 38 newly collected *Capnodiaceae* strains were examined in this study. These strains were associated with 36 host plant taxa belonging to 17 genera and 16 families and were collected from several provinces in China, with most collections originating from Guizhou Province. The host plants and geographic sources of the newly collected taxa are summarized in Table 3.

**Table 3.** Host plants and geographic distribution of newly collected *Capnodiaceae* taxa in this study.

Taxon	Strain	Host plant	Host family	Province/region	Record significance
*Conidiocarpus cinnamomi*	IFRDCC 25-0005	*Forsythia suspensa*	* Oleaceae *	Guizhou Province	New provincial record for Guizhou; first report from *Forsythia suspensa*
*Conidiocarpus cinnamomi*	IFRDCC 19-1150	*Eriobotrya* sp.	* Rosaceae *	Guizhou Province	New provincial record for Guizhou; first report from *Eriobotrya* sp.
*Conidiocarpus cinnamomi*	IFRDCC 19-1174	*Lauraceae* sp.	* Lauraceae *	Guizhou Province	New provincial record for Guizhou; first report from *Lauraceae* sp.
*Conidiocarpus cinnamomi*	IFRDCC 19-1168	*Viburnum* sp.	* Adoxaceae *	Guizhou Province	New provincial record for Guizhou; first report from *Viburnum* sp.
*Conidiocarpus cinnamomi*	IFRDCC 19-1165	*Phyllostachys* sp.	* Poaceae *	Guizhou Province	New provincial record for Guizhou; first report from *Phyllostachys* sp.
*Conidiocarpus cinnamomi*	IFRDCC 19-1171	*Eriobotrya* sp.	* Rosaceae *	Guizhou Province	New provincial record for Guizhou; first report from *Eriobotrya* sp.
*Conidiocarpus luodianense*	IFRDCC 19-1153	*Iris* sp.	* Iridaceae *	Guizhou Province	Additional material of new species
*Conidiocarpus luodianense*	IFRDCC 19-1183	*Citrus maxima*	* Rutaceae *	Guizhou Province	New species
*Conidiocarpus luodianense*	IFRDCC 19-1177	*Gardenia* sp.	* Rubiaceae *	Guizhou Province	Additional material of new species
*Conidiocarpus bijieense*	IFRDCC 19-1189	*Ficus* sp.	* Moraceae *	Guizhou Province	New species
*Conidiocarpus bijieense*	IFRDCC 19-1186	*Ficus* sp.	* Moraceae *	Guizhou Province	Additional material of new species
*Conidiocarpus caucasicus*	IFRDCC 25-0006	*Viburnum utile*	* Adoxaceae *	Guizhou Province	New provincial record for Guizhou; first report from *Viburnum utile*
*Conidiocarpus caucasicus*	IFRDCC 19-1195	*Viburnum* sp.	* Adoxaceae *	Guizhou Province	New provincial record for Guizhou; first report from *Viburnum* sp.
*Conidiocarpus caucasicus*	IFRDCC 19-1192	*Citrus maxima*	* Rutaceae *	Guizhou Province	New provincial record for Guizhou; first report from *Citrus maxima*
*Conidiocarpus caucasicus*	IFRDCC 19-1159	*Osmanthus* sp.	* Oleaceae *	Guizhou Province	New provincial record for Guizhou; first report from *Osmanthus* sp.
*Conidiocarpus tainingense*	IFRDCC 19-1198	*Camellia* sp.	* Theaceae *	Fujian Province	New species
*Conidiocarpus guilanensis*	IFRDCC 25-0007	*Rubus lambertianus*	* Rosaceae *	Guizhou Province	New record for China
*Conidiocarpus guilanensis*	IFRDCC 19-1213	*Ficus* sp.	* Moraceae *	Guizhou Province	New record for China
*Hyphopolychaeton duyunense*	IFRDCC 25-0011	*Laurus nobilis*	* Lauraceae *	Guizhou Province	New genus and new species; type species of *Hyphopolychaeton*
*Hyphopolychaeton duyunense*	IFRDCC 25-0013	*Heptapleurum rigidum*	* Araliaceae *	Guizhou Province	Additional material of new species
*Hyphopolychaeton duyunense*	IFRDCC 25-0014	*Rubus lambertianus*	* Rosaceae *	Guizhou Province	Additional material of new species
*Hyphopolychaeton duyunense*	IFRDCC 25-0008	*Eurya chinensis*	* Pentaphylacaceae *	Guizhou Province	Additional material of new species
*Hyphopolychaeton duyunense*	IFRDCC 25-0015	*Corylopsis stelligera*	* Hamamelidaceae *	Guizhou Province	Additional material of new species
*Hyphopolychaeton duyunense*	IFRDCC 25-0010	*Corylopsis stelligera*	* Hamamelidaceae *	Guizhou Province	Additional material of new species
*Hyphopolychaeton duyunense*	IFRDCC 25-0009	*Eurya chinensis*	* Pentaphylacaceae *	Guizhou Province	Additional material of new species
*Hyphopolychaeton duyunense*	IFRDCC 25-0012	*Ampelocalamus naibunensi*s	* Poaceae *	Guizhou Province	Additional material of new species
*Hyphopolychaeton duyunense*	IFRDCC 25-0016	*Lithocarpus corneus*	* Fagaceae *	Guizhou Province	Additional material of new species
*Hyphopolychaeton cengongense*	IFRDCC 25-0020	*Lagerstroemia indica*	* Lythraceae *	Guizhou Province	New species
*Hyphopolychaeton cengongense*	IFRDCC 25-0018	*Dalbergia assamica*	* Fabaceae *	Guizhou Province	Additional material of new species
*Hyphopolychaeton cengongense*	IFRDCC 25-0019	*Lagerstroemia indica*	* Lythraceae *	Guizhou Province	Additional material of new species
*Hyphopolychaeton cengongense*	IFRDCC 25-0017	*Alpinia nutans*	* Zingiberaceae *	Guangdong Province	Additional material of new species
*Hyphopolychaeton maolanense*	IFRDCC 25-0021	*Smilax ocreata*	* Smilacaceae *	Guizhou Province	New species
*Capnodium neocoffeicola*	IFRDCC 19-1201	*Ficus* sp.	* Moraceae *	Guizhou Province	New record for China; first isolation from *Ficus* sp.
*Polychaeton citruscola*	IFRDCC 19-1216	*Citrus maxima*	* Rutaceae *	Guizhou Province	New species
*Leptoxyphium fumago*	IFRDCC 19-1219	*Maling bamboo*	* Poaceae *	Guizhou Province	New record for China; first report from *Maling bamboo*
*Leptoxyphium chishuiense*	IFRDCC 19-1222	*Alsophila* sp.	* Cyatheaceae *	Guizhou Province	New species
*Leptoxyphium chishuiense*	IFRDCC 19-1225	*Maling bamboo*	* Poaceae *	Zhejiang Province	Additional material of new species

Host identity was assessed using plant morphological information and, where possible, the *rbcL* sequence data. Reliable *rbcL* sequences were obtained for 14 host plant species, and the corresponding host molecular identification data are provided in Suppl. material 1. For samples for which *rbcL* amplification was unsuccessful, host identification was based mainly on morphological evidence.

Several taxa were recovered from more than one host plant, including *Conidiocarpus cinnamomi*, *C. caucasicus*, *Hyphopolychaeton duyunense*, and *Hyphopolychaeton cengongense*. These records indicate that some *Capnodiaceae* taxa are not restricted to a single host species but may occur on different plant hosts under suitable subtropical conditions. However, host association was not used as a primary criterion for species delimitation in the present study. Instead, host and geographic information were interpreted together with morphology and multilocus phylogenetic evidence to document the ecological breadth and regional distribution of the examined taxa.

### Taxonomy

#### Fungi

***Ascomycota* Caval. Sm**.

***Dothideomycetes* O. E. Erikss. & Winka**.

***Capnodiales* Woron., Annls mycol. 23(1/2): 177 (1925)**.

***Capnodiaceae* Höhn. ex Theiss., Verh. zool.-Bot. Ges. Wien 66: 363 (1916)**.

### Key to the genera of *Capnodiaceae* represented in this study

**Table:** 

1	Sexual morph unknown or not observed	**2**
–	Sexual morph known, ascomata present	**3**
2	Hyphomycetous; conidia brown, septate, in acropetal chains	*** Heteroconium ***
–	Synnemata present	**4**
–	Pycnidial or pycnidium-like conidiomata present	**5**
3	Asci 8-spored, ascospores mostly 3-septate	*** Chaetocapnodium ***
–	Asci 8-spored, ascospores mostly 4-septate	*** Phragmocapnias ***
4	Conidia subcylindrical to fusiform	*** Hyphocapnodia ***
5	Conidiomata with upper-middle swollen region	*** Conidiocarpus ***
–	Conidiomata with middle-lower swollen region	**6**
–	Conidiomata with swollen region at apex	**7**
6	Conidiomata with a distinct basal part	*** Capnodium ***
–	Conidiomata lacking a distinct basal part	*** Polychaeton ***
7	Apex cup-shaped, producing conidia	*** Leptoxyphium ***
–	Apex ellipsoid, producing conidia, lacking a long neck	*** Hyphopolychaeton ***

#### Conidiocarpus

Taxon classification

Animalia

CapnodialesCapnodiaceae

Woron., Ann. Mycol. 24: 250 (1927)

A805B485-3AE7-5E7A-9C52-72449601FFED

##### Type species.

*Conidiocarpus caucasicus* Woron., Key to fungi (fungi imperfecti) 2: 743 (1917).

##### Notes.

*Conidiocarpus* was previously regarded as the asexual morph of *Phragmocapnias* and was treated as a synonym of *Phragmocapnias* by earlier authors (Hughes 1976; Chomnunti et al. 2011; Chomnunti et al. 2014; Hongsanan et al. 2015). However, Abdollahzadeh et al. (2020) treated *Conidiocarpus* and *Phragmocapnias* as separate genera based on morphology and multilocus phylogeny, and this treatment has been followed in subsequent phylogenetic studies. Based on recent taxonomic and phylogenetic studies, *Conidiocarpus* currently includes 16 named species (Abdollahzadeh et al. 2020; Khodaparast et al. 2020; Tennakoon et al. 2021; Pem et al. 2024; Yang et al. 2025; Sun et al. 2026). Species represented by molecular data are mainly interpreted according to their placement as independent lineages in multilocus phylogenetic analyses, together with diagnostic morphological characters. Names lacking molecular data are retained according to their original morphological descriptions and nomenclatural treatments, but their phylogenetic positions remain unconfirmed until sequence data become available. *Conidiocarpus* is distinguished from other genera by pycnidial conidiomata with the swollen conidiogenous region located in the upper-middle part of the conidioma, usually accompanied by an elongated basal part and a long stalk (Chomnunti et al. 2011; Abdollahzadeh et al. 2020).

#### Conidiocarpus cinnamomi

Taxon classification

Animalia

CapnodialesCapnodiaceae

X.Y. Li & C.L. Yang Mycosphere 16(1): 1882 (2025)

6E754047-D76A-5618-937A-F8F084094C90

Fig. 2

##### Description.

***Epiphytic*** on the leaf surface of *Forsythia suspensa*, forming a sooty coating on adaxial surface. Thallus composed of brown, septate, ellipsoidal, smooth-walled hyphae. ***Asexual morph. Conidiomata*** (282–398 × 25–53 µm, x̄ = 375 × 39 µm, *n* = 20) long, pycnidial, elongate, superficial, stipitate, with a ***long*-*stalk*** (138–275 µm, x̄ = 194 µm, *n* = 15), black, rigid, a distinct ***neck*** (62–126 µm, x̄ = 96 µm, *n* = 20) height, and a prominently ovoid-swollen conidiogenous region, the swollen area producing conidia inside. ***Conidiomata apex*** gradually light brown to hyaline, with a circular ***ostiole*** (7–16 µm, x̄ = 12 µm, *n* = 20) diam, consisting of rectangular, compact cells, surrounded by hyaline hyphae. ***Conidiogenous region*** (22–39 µm, x̄ = 32 µm, *n* = 20) wide, located in the upper-middle part of the conidiomata, brown, composed of cylindrical, thin-walled cells. ***Conidia*** (4–5.5 × 2.1–3.9 μm, x̄ = 4.5 × 2.5 µm, *n* = 30), hyaline, single-celled, ellipsoidal, smooth-walled, guttulate with 1–2 distinct refractive oil droplets, exuding in creamy masses from the ostiole. ***Sexual morph***. Undetermined.

**Figure 2.**
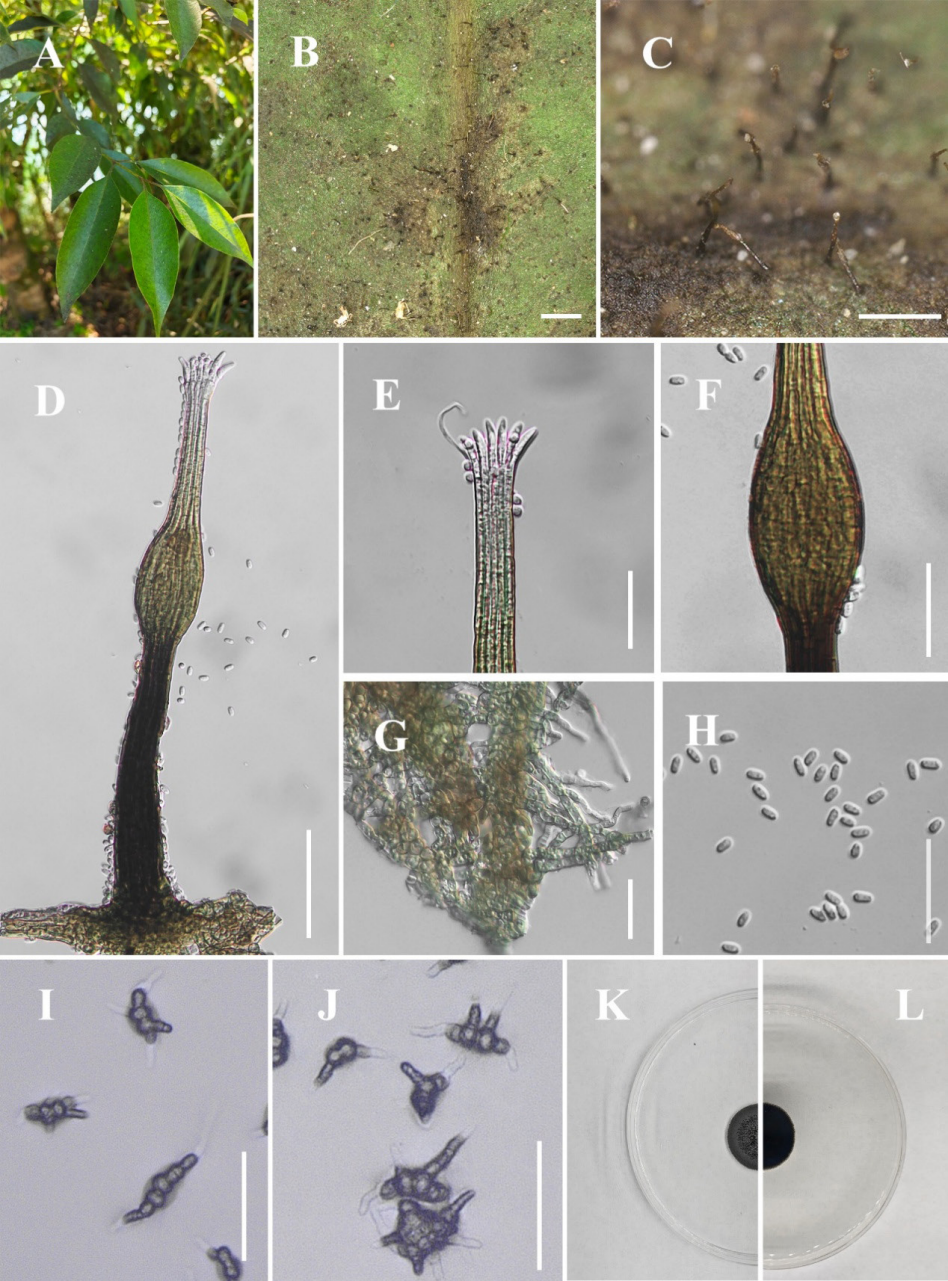
*Conidiocarpus cinnamomi* (IFRDCC 25-0005). **A** Black mycelium covering the leaf surface; **B, C** Conidiomata on host surface; **D** Conidioma; **E** Ostiolar neck; **F** Prominently ovoid-swollen conidiogenous region; **G** Mycelial network; **H** Conidia; **I, J** Germinated conidia; **K** Front view of the colony on PDA; **L** Reverse view of the colony on PDA. Scale bars: 500 μm (**B**); 250 μm (**C**); 50 μm (**D, I, J**); 25 μm (**E–H**).

##### Culture characteristics.

Colonies growing on PDA reaching 22.63 mm in diam after 14 days at 26 °C in the dark, colony surface gradually erumpent, with hyphae growing downward and immersed in the medium, olivaceous.

##### Material examined.

China • Guizhou Province, Huaxi District, Huaxi Park, on living leaves of *Forsythia suspensa* (26°27'03"N, 106°40'30"E), 24 August 2025, Jipeng Sun, HX-4 (IFRD 900-12), living culture IFRDCC 25-0005.

##### Notes.

In the phylogenetic tree, six newly collected strains of *Conidiocarpus cinnamomi* (IFRDCC 25-0005, IFRDCC 19-1150, IFRDCC 19-1174, IFRDCC 19-1168, IFRDCC 19-1165, and IFRDCC 19-1171) clustered with *C. cinnamomi* SICAU 23-0100 in a single clade with BS/PP support values of 95%/0.93. Molecular sequence comparison revealed no nucleotide differences between *C. cinnamomi* (IFRDCC 25-0005) and *C. cinnamomi* (SICAU 23-0100). However, slight morphological differences were observed between *C. cinnamomi* (IFRDCC 25-0005) and *C. cinnamomi* (SICAU 23-0100), particularly in conidiomatal size, neck length, and ostiole dimensions. Because these differences were not accompanied by molecular divergence in the examined loci, they are interpreted here as intraspecific variation rather than evidence for species-level separation. Similar minor quantitative variation has also been reported in other *Capnodiaceae* taxa (Abdollahzadeh et al. 2020; Thungdee et al. 2023; Tian et al. 2024), suggesting that such size-related characters should be interpreted cautiously in species delimitation. Accordingly, the strains examined here are identified as the known species *C. cinnamomi*.

In this study, the six newly collected strains of *C. cinnamomi* were isolated from six collected specimens representing five host taxa, namely *Forsythia suspensa*, *Eriobotrya* sp., *Lauraceae* sp., *Viburnum* sp., and *Phyllostachys* sp. These collections represent the first record of *C. cinnamomi* from Guizhou Province, China, and expand the known host range of this species. This study also provides ITS, LSU, *tef*1-α, and *rpb*2 sequence data for all examined strains, together with corresponding host information.

#### Conidiocarpus luodianense

Taxon classification

Animalia

CapnodialesCapnodiaceae

J.P. Sun & X.Y. Zeng sp. nov.

B8D7CE92-835A-5E32-9D6E-DC452E880E55

Index Fungorum: IF904322

Fig. 3

##### Etymology.

This name refers to the location of “Luodian County,” where the complete specimens were collected.

**Figure 3.**
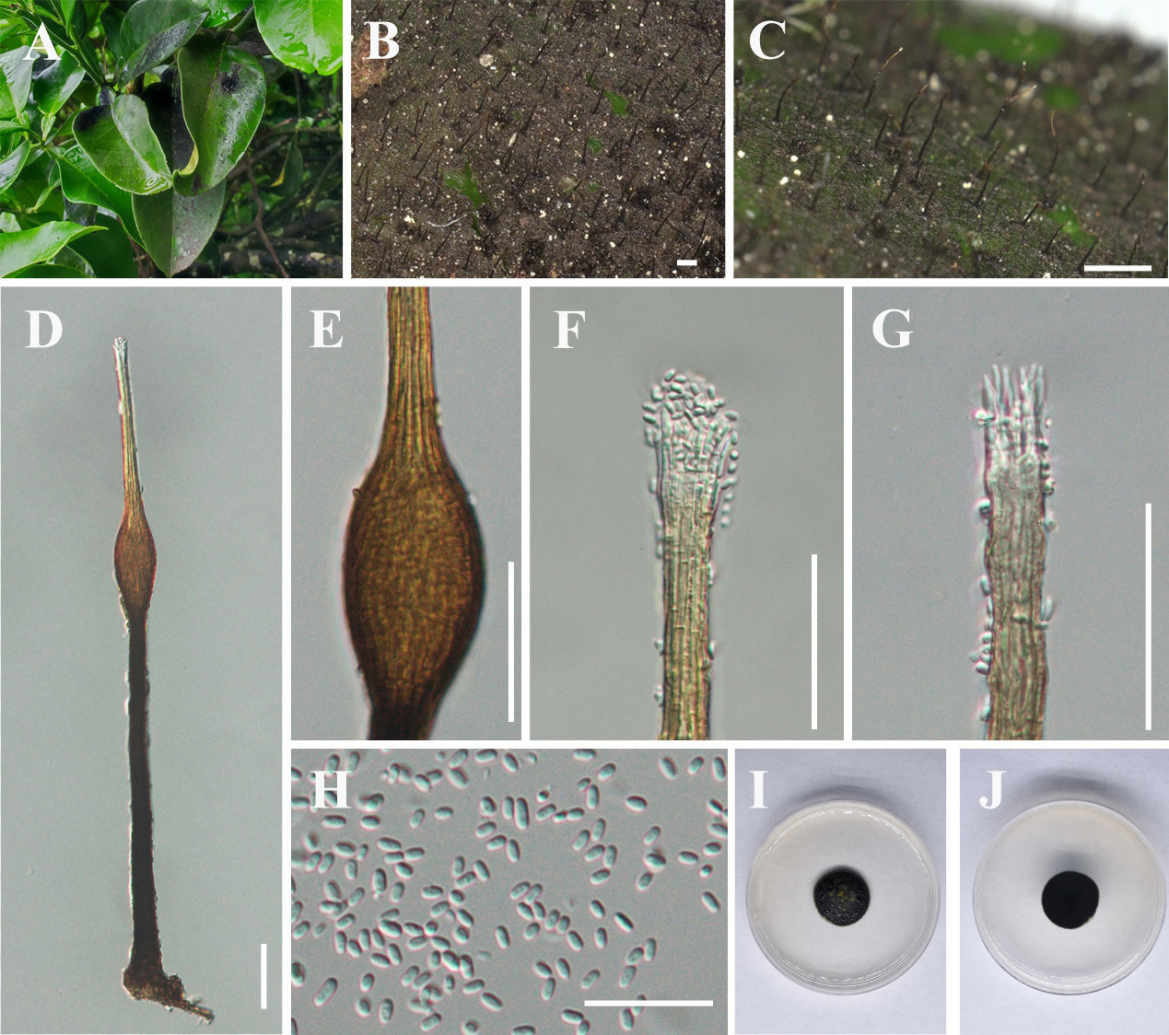
*Conidiocarpus luodianense* (IFRD 99011, holotype). **A** Black mycelium covering the leaf surface; **B, C** Conidiomata on host surface; **D** Conidioma; **E** Prominently ovoid-swollen conidiogenous region; **F, G** Ostiolar neck; **H** Conidia; **I** Front view of the colony on PDA; **J** Reverse view of the colony on PDA. Scale bars: 200 μm (**B**); 500 μm (**C**); 50 μm (**D–G**); 25 μm (**H**).

##### Diagnosis.

Differs from *Conidiocarpus cinnamomi* X.Y. Li & C.L. Yang by significantly shorter conidiomata, narrower bases with conspicuously wider ostioles, and smaller conidia. Differs from *Conidiocarpus tainingense* J.P. Sun & X.Y. Zeng by significantly narrow bases and smaller conidia.

##### Holotype.

IFRD 99011.

##### Description.

***Epiphytic*** on the leaf surface of *Citrus maxima*, forming a sooty coating on adaxial surface. Thallus composed of brown, septate, ellipsoidal, smooth-walled hyphae. ***Asexual morph. Conidiomata*** (487–527 × 10–18 µm, x̄ = 507 × 14 µm, *n* = 10) long, pycnidial, elongate, superficial, stipitate, with a ***long*-*stalk***, black, rigid, a distinct ***neck***, and a prominently ovoid-swollen conidiogenous region, the swollen area producing conidia inside. ***Conidiogenous region*** (32–38 µm, x̄ = 35 µm, *n* = 10) wide, located in the upper-middle part of the conidiomata, brown, composed of cylindrical, thin-walled cells. ***Conidiomata apex*** gradually light brown to hyaline, with a circular ***ostiole*** (8.5–10.1 µm, x̄ = 9.2 µm, *n* = 10) diam, consisting of rectangular, compact cells, surrounded by hyaline hyphae. ***Conidia*** (2.6–3.8 × 1.6–2 µm, x̄ = 3.2 × 1.8 µm, *n* = 30), hyaline, single-celled, ellipsoidal, smooth-walled, guttulate without distinct refractive oil droplet, exuding in creamy masses from the ostiole. ***Sexual morph***. Undetermined.

##### Culture characteristics.

Colonies slow growing, reaching 19.8 mm diam after 14 days at 26 °C in darkness on PDA, colony superficial to gradually erumpent, with hyphae growing downward and fine, downy filaments immersed in medium, olivaceous.

##### Material examined.

China • Guizhou Province, Luodian County, on living leaves of *Citrus maxima* (25°21'38"N, 106°30'36"E), 14 May 2022, Yuwei Liu, BY-5 (IFRD 99011, holotype), ex-type living culture IFRDCC 19-1183.

##### Notes.

In the phylogenetic tree, *Conidiocarpus luodianense* is represented by three strains (IFRDCC 19-1153, IFRDCC 19-1177, and IFRDCC 19-1183) and forms a distinct lineage close to *C. cinnamomi*, with BS/PP support values of 98%/0.99. *Conidiocarpus nanshanense* is resolved in a neighboring lineage, whereas *C. luodianense* remains distinct. Among the examined loci, *rpb*2 provides the strongest molecular signal for separating *C. luodianense* from *C. cinnamomi*. Pairwise sequence comparisons between *C. luodianense* (IFRDCC 19-1183) and *C. cinnamomi* (SICAU 23-0100) showed 98.43% similarity in *rpb*2 (819/832 bp, 7 gaps), 99.14% similarity in ITS (462/466 bp, 1 gap), and no nucleotide differences in LSU and *tef*1-α. However, the recognition of *C. luodianense* is not based on *rpb*2 alone. Morphologically, *C. luodianense* differs from *C. cinnamomi* mainly in having markedly narrower bases (10–18 μm vs. 26–47 μm), smaller conidia (3.2 × 1.8 μm vs. 5.5 × 2.8 μm), wider ostioles (8.5–10.1 μm vs. 4–8 μm), and generally shorter conidiomata (487–527 μm vs. 500–860 μm) (Yang et al. 2025). It also differs from *C. nanshanense* in having markedly narrower bases (10–18 μm vs. 27–53 μm) and smaller conidia (2.6–3.8 × 1.6–2 μm vs. 4.1–6.4 × 2.4–3.3 μm) (Sun et al. 2026). These differences, together with its independent phylogenetic placement, support the recognition of *C. luodianense* as a distinct species.

#### Conidiocarpus bijieense

Taxon classification

Animalia

CapnodialesCapnodiaceae

J.P. Sun & X.Y. Zeng sp. nov.

98D03EE3-68DC-5144-A7D1-A5BD1ED87B27

Index Fungorum: IF905003

Fig. 4

##### Etymology.

This name refers to the location of “Bijie City,” where the complete specimens were collected.

**Figure 4.**
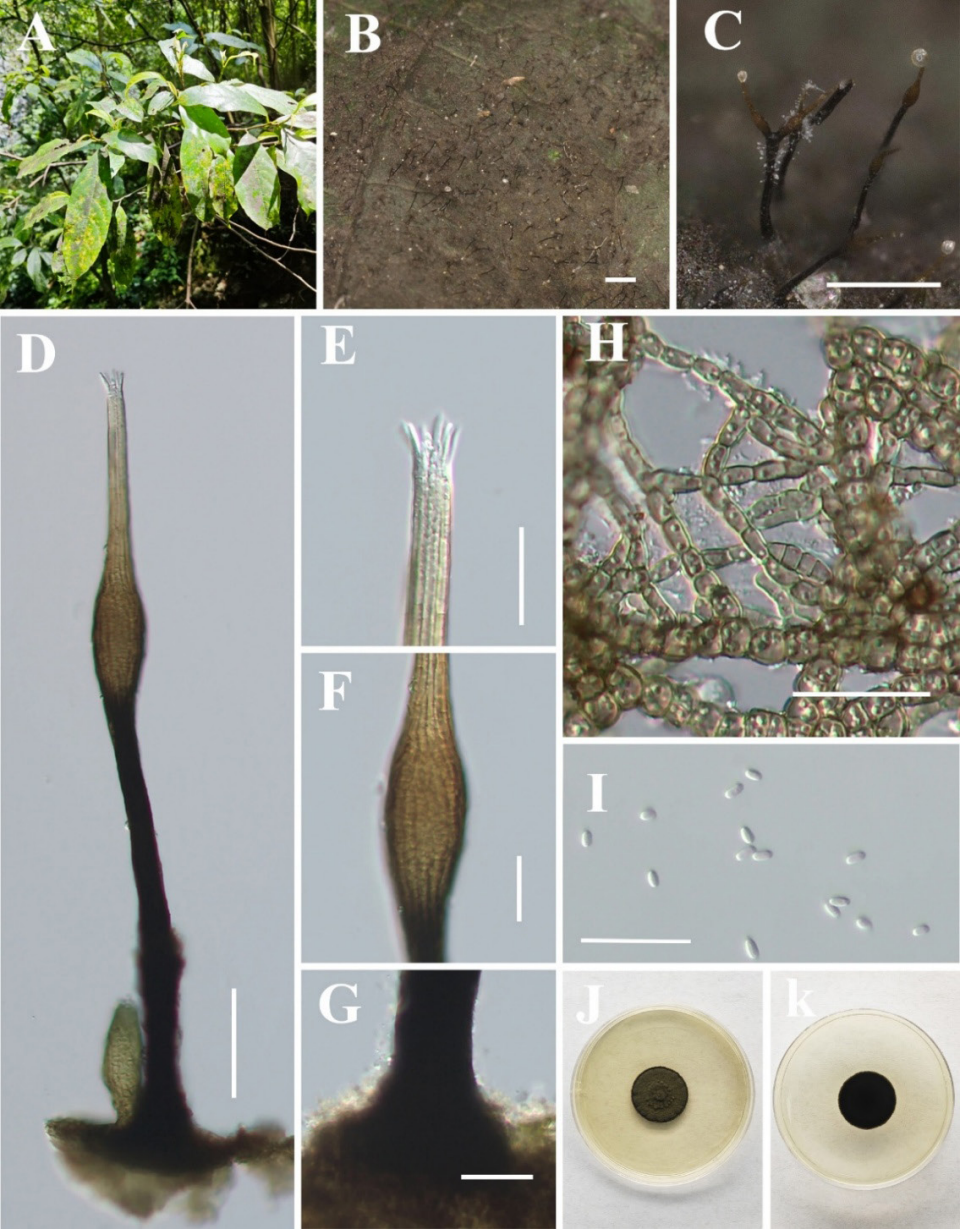
*Conidiocarpus bijieense* (IFRD 99013, holotype). **A** Black mycelium covering the leaf surface; **B, C** Conidiomata on the host; **D** Conidioma; **E** Ostiolar neck; **F** Prominently ovoid-swollen conidiogenous region; **G** Base of conidioma; **H** Mycelial network; **I** Conidia; **J** Front view of the colony on PDA; **K** Reverse view of the colony on PDA. Scale bars: 500 μm (**B**); 250 μm (**C**); 50 μm (**D**); 20 μm (**E–I)**.

##### Diagnosis.

Differs from *Conidiocarpus cinnamomi* X.Y. Li & C.L. Yang by significantly smaller conidiomata, narrower bases, wider ostioles, narrower conidiogenous, and smaller conidia. Differs from *Conidiocarpus luodianense* J.P. Sun & X.Y. Zeng by significantly smaller conidiomata and narrower conidiogenous.

##### Holotype.

IFRD 99013.

##### Description.

***Epiphytic*** on the leaf surface of *Ficus sp*., forming a sooty coating on adaxial surface. Thallus composed of brown, septate, ellipsoidal, smooth-walled hyphae. ***Asexual morph. Conidiomata*** (380–420 × 14–22 µm, x̄ = 400 × 18 µm, *n* = 10) long, pycnidial, elongate, superficial, stipitate, with a ***long*-*stalk***, black, rigid, a distinct ***neck***, and a prominently ovoid-swollen conidiogenous region, the swollen area producing conidia inside. ***Conidiomata apex*** gradually light brown to hyaline, with a circular ***ostiole*** (9.7–11.3 µm, x̄ = 10.4 µm, *n* = 10) diam, consisting of rectangular, compact cells, surrounded by hyaline hyphae. ***Conidiogenous region*** (27–33 µm, x̄ = 30 µm, *n* = 10) wide, located in the upper-middle part of the conidiomata, brown, composed of cylindrical, thin-walled cells. ***Conidia*** (2.6–3.8 × 1.6–2 µm, x̄ = 3.2 × 1.8 µm, *n* = 30), hyaline, single-celled, ellipsoidal, smooth-walled, guttulate with 1–2 distinct refractive oil droplets, exuding in creamy masses from the ostiole. ***Sexual morph***. Undetermined.

##### Culture characteristics.

Colonies slow growing, reaching 21.62 mm diam after 14 days at 26 °C in darkness on PDA, colony superficial to gradually erumpent, with hyphae growing downward and immersed in medium, olivaceous.

##### Material examined.

China • Guizhou Province, Bijie City, on living leaves of *Ficus* sp. (26°57'35"N, 105°20'19"E), 23 July 2023, Yuwei Liu, BJ-2 (IFRD 99013, holotype), ex-type living culture IFRDCC 19-1189.

##### Notes.

In the phylogenetic tree, *Conidiocarpus bijieense* is represented by two strains (IFRDCC 19-1189 and IFRDCC 19-1186) and forms a distinct lineage close to *C. cinnamomi* (SICAU 23-0100) and *C. luodianense* (IFRDCC 19-1183), with BS/PP support values of 94%/0.96. After the inclusion of the recently described *C. nanshanense* (IFRDCC 25-0001), *C. bijieense* remains distinct from *C. cinnamomi*, *C. luodianense*, and the neighboring *C. nanshanense* lineage. The ex-type living culture of *C. bijieense* (IFRDCC 19-1189) yielded sequence data for ITS, LSU, and *tef*1-α, but not for *rpb*2. Therefore, comparison of the *rpb*2 locus was supplemented using *C. bijieense* (IFRDCC 19-1186), which belongs to the same lineage. Compared with *C. cinnamomi* (SICAU 23-0100), *C. bijieense* showed the strongest molecular differentiation in *tef*1-α (98.47%, 770/782 bp, 0 gaps), followed by *rpb*2 (99.77%, 875/877 bp, 0 gaps) and ITS (99.78%, 453/454 bp, 1 gap), whereas LSU showed no nucleotide differences. Compared with *C. luodianense* (IFRDCC 19-1183), *C. bijieense* showed differences in *tef*1-α (98.63%, 864/876 bp, 0 gaps) and *rpb*2 (98.86%, 957/968 bp, 7 gaps), whereas ITS and LSU showed no nucleotide differences. These results indicate that the distinction of *C. bijieense* is supported mainly by protein-coding loci, especially *tef*1-α and *rpb*2, rather than by ribosomal markers alone. Morphologically, *C. bijieense* (IFRDCC 19-1189) differs from *C. cinnamomi* (SICAU 23-0100) in having smaller conidiomata (380–420 μm vs. 500–860 μm), narrower bases (14–22 μm vs. 26–47 μm), wider ostioles (9.7–11.3 μm vs. 4–8 μm), narrower conidiogenous regions (27–33 μm vs. 37–57 μm), and smaller conidia (2.6–3.8 × 1.6–2 μm vs. 4.5–5.2 × 1.9–2.8 μm) (Yang et al. 2025). It differs from *C. luodianense* (IFRDCC 19-1183) in having smaller conidiomata (380–420 μm vs. 487–527 μm) and narrower conidiogenous regions (27–33 μm vs. 32–38 μm) (this study, above). It also differs from *C. nanshanense* (IFRDCC 25-0001) in having narrower bases (14–22 μm vs. 27–53 μm) and smaller conidia (2.6–3.8 × 1.6–2 μm vs. 4.1–6.4 × 2.4–3.3 μm) (Sun et al. 2026). These stable morphological differences, together with its independent phylogenetic placement and protein-coding gene differentiation, support the recognition of *C. bijieense* as a distinct species.

#### Conidiocarpus caucasicus

Taxon classification

Animalia

CapnodialesCapnodiaceae

Woron. Key to fungi (fungi imperfecti) 2: 743 (1917).

F4D7CF1C-135E-5509-8178-7468E6D365B9

Fig. 5

##### Description.

***Epiphytic*** on the leaf surface of *Viburnum utile*, forming a sooty coating on adaxial surface. Thallus composed of brown, septate, ellipsoidal, smooth-walled hyphae. ***Asexual morph. Conidiomata*** (390–576 × 34–84 µm, x̄ = 486 × 47 µm, *n* = 20) long, pycnidial, elongate, superficial, stipitate, with a ***long*-*stalk*** (173–357 µm, x̄ = 266 µm, *n* = 20), black, rigid, a distinct ***neck*** (101–157 µm, x̄ = 124 µm, *n* = 20) height, and a prominently ovoid-swollen conidiogenous region, the swollen area producing conidia inside. ***Conidiomata apex*** gradually light brown to hyaline, with a circular ***ostiole*** (10–18 µm, x̄ = 12 µm, *n* = 20) diam, consisting of rectangular, compact cells, surrounded by hyaline hyphae. ***Conidiogenous region*** (30–47 µm, x̄ = 38 µm, *n* = 20) wide, located in the upper-middle part of the conidiomata, brown, composed of cylindrical, thin-walled cells. ***Conidia*** (4–5.7 × 2.4–3.3 µm, x̄ = 5.2 × 2.9 µm, *n* = 30), hyaline, single-celled, ellipsoidal, smooth-walled, guttulate with 1–2 distinct refractive oil droplets, exuding in creamy masses from the ostiole. ***Sexual morph***. Undetermined.

**Figure 5.**
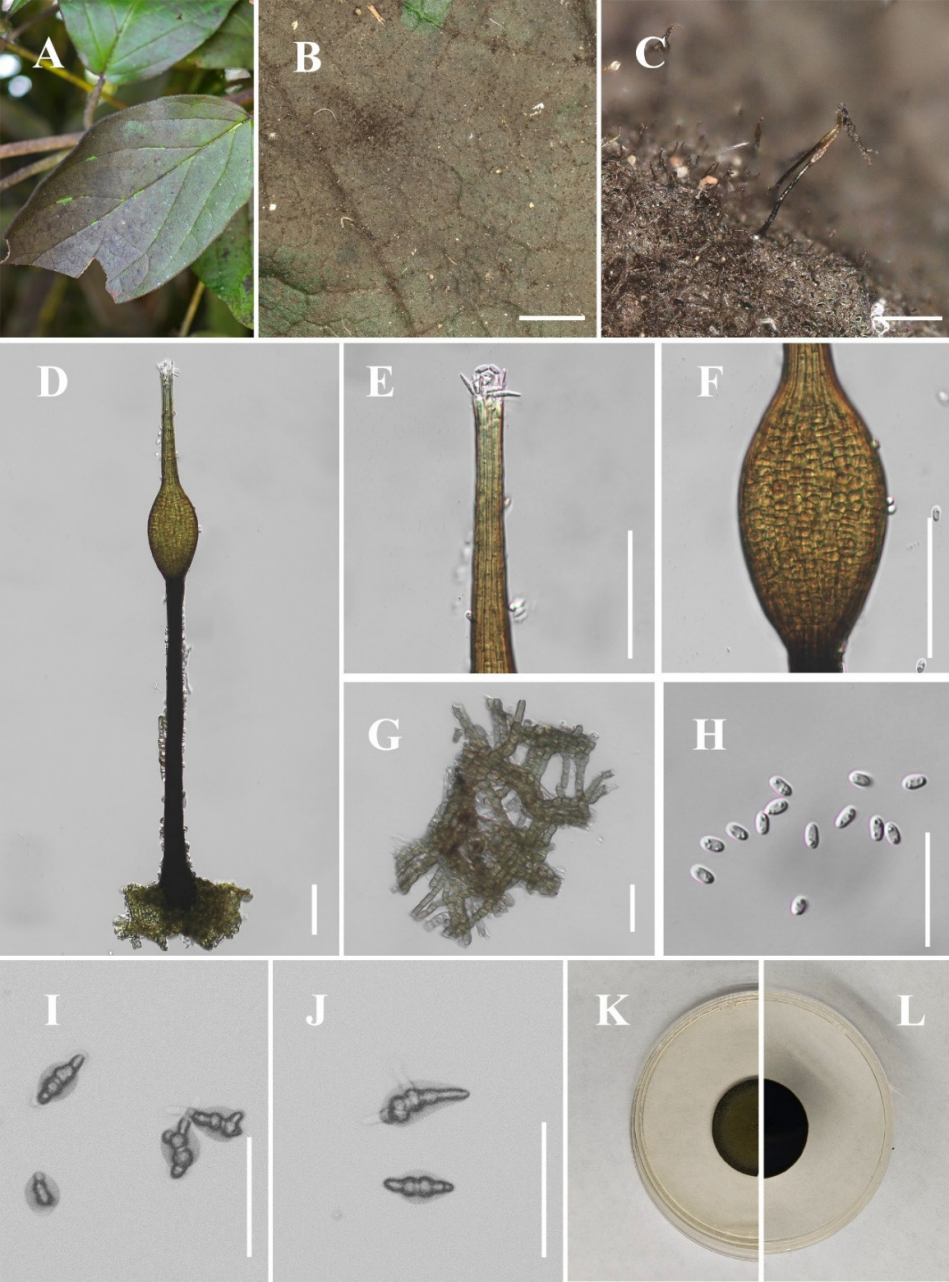
*Conidiocarpus caucasicus* (IFRDCC 25-0006). **A** Black mycelium covering the leaf surface; **B, C** Conidiomata on host; **D** Conidioma; **E** Ostiolar neck; **F** Prominently ovoid-swollen conidiogenous region; **G** Mycelial network; **H** Conidia; **I, J** Germinated conidia; **K** Front view of the colony on PDA; **L** Reverse view of the colony on PDA. Scale bars: 1000 μm (**B**); 100 μm (**C**); 50 μm (**D–F**); 25 μm (**G, H**); 100 μm (**I, J**).

##### Culture characteristics.

Colonies slow growing, reaching 21.61 mm diam after 14 days at 26 °C in darkness on PDA, colony superficial to gradually erumpent, with hyphae growing downward and immersed in medium, olivaceous.

##### Material examined.

China • Guizhou Province, Guiyang City, Huaxi Park, on living leaves of *Viburnum utile* (26°27'03"N, 106°40'30"E), 31 August 2025, Jipeng Sun, HX-3 (IFRD 900-13), living culture IFRDCC 25-0006.

##### Notes.

In the phylogenetic tree, the four strains of *Conidiocarpus caucasicus* obtained in this study (IFRDCC 19-1195, IFRDCC 25-0006, IFRDCC 19-1192, and IFRDCC 19-1159) clustered together with *C. caucasicus* (UESTCC 23.0246) in a single clade, with BS/PP support values of 93%/0.99. *C. caucasicus* (GUMH 937) is historically important as reference material of *C. caucasicus* (Khodaparast 2006), but within the four-locus dataset used in the present study, only LSU is directly comparable for this material. Pairwise LSU comparison between *C. caucasicus* (GUMH 937) and *C. caucasicus* (UESTCC 23.0246) showed high similarity (99.88%, 808/809 bp, 0 gaps). However, LSU also showed similarly high similarities among several closely related *Conidiocarpus* taxa in this clade, indicating limited species-level resolution. Therefore, *C. caucasicus* (UESTCC 23.0246), which was provided with morphology and ITS, LSU, *tef*1-α, and *rpb*2 sequence data by Tian et al. (2024), was used here as the directly comparable reference culture for interpreting the newly collected Guizhou strains in the present multilocus framework. The Guizhou strains are phylogenetically consistent with *C. caucasicus* (UESTCC 23.0246) and are therefore treated as *C. caucasicus*. Minor morphological differences observed between the present collections and previously published material are interpreted as intraspecific variation.

#### Conidiocarpus tainingense

Taxon classification

Animalia

CapnodialesCapnodiaceae

J.P. Sun & X.Y. Zeng sp. nov.

368A9CC1-E563-59B7-8B66-C8CCED20567E

Index Fungorum: IF904318

Fig. 6

##### Etymology.

This name refers to the location of “Taining County,” where the complete specimens were collected.

**Figure 6.**
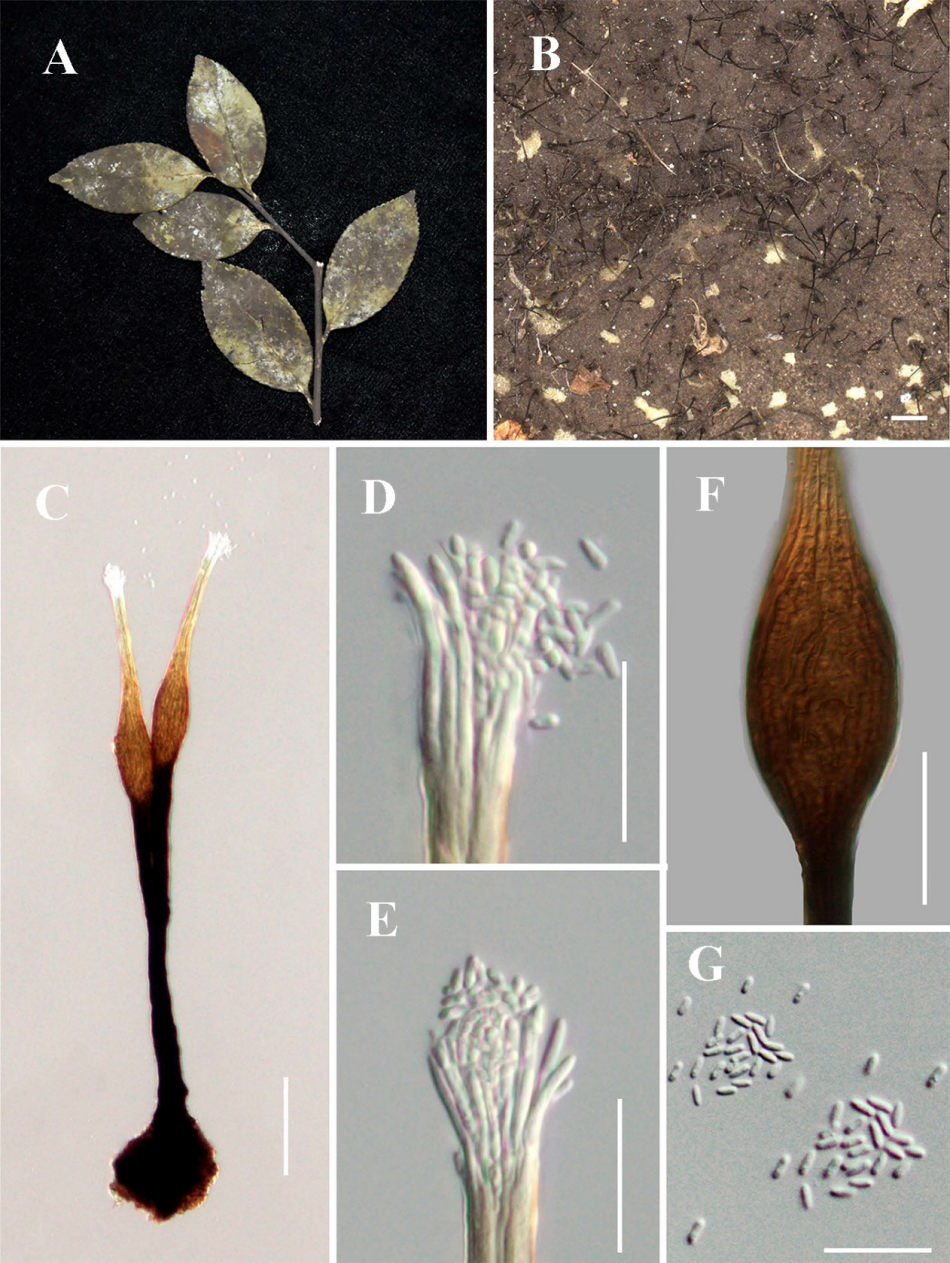
*Conidiocarpus tainingense* (IFRD 900-24, holotype). **A**, **B** Black mycelium covering the leaf surface; **C** Conidioma; **D**, **E** Ostiole; **F** Prominently ovoid-swollen conidiogenous region; **G** Conidia. Scale bars: 500 μm (**B**); 100 μm (**C**); 25 μm (**D, E, G**); 50 μm (**F**).

##### Diagnosis.

Differs from *Conidiocarpus caucasicus* Woron. by significantly narrower bases, ostioles, and conidiogenous. Differs from *Conidiocarpus cinnamomi* X.Y. Li & C.L. Yang by significantly smaller conidiomata, wider ostioles, and narrower conidiogenous.

##### Holotype.

IFRD 900-24.

##### Description.

***Epiphytic*** on the leaf surface of *Camellia* sp., forming a sooty coating on adaxial surface. Thallus composed of brown, septate, ellipsoidal, smooth-walled hyphae. ***Asexual morph. Conidiomata*** (424–504 × 23–32 µm, x̄ = 454 × 26 µm, *n* = 10) long, pycnidial, elongate, superficial, stipitate, with a ***long*-*stalk***, black, rigid, a distinct ***neck***, and a prominently ovoid-swollen conidiogenous region, the swollen area producing conidia inside. ***Conidiomata apex*** gradually light brown to hyaline, with a circular ***ostiole*** (7–13 µm, x̄ = 10 µm, *n* = 10) diam, consisting of rectangular, compact cells, surrounded by hyaline hyphae. ***Conidiogenous region*** (29–34 µm, x̄ = 31 µm, *n* = 10) wide, located in the upper-middle part of the conidiomata, brown, composed of cylindrical, thin-walled cells. ***Conidia*** (4.8–5.5 × 1.7–2.2 μm, x̄ = 5.1 × 1.9 μm, *n* = 30), hyaline, single-celled, ellipsoidal, smooth-walled, guttulate without distinct refractive oil droplet, exuding in creamy masses from the ostiole. ***Sexual morph***. Undetermined.

##### Culture characteristics.

Colonies slow growing, reaching 11.13 mm diam after 14 days at 26 °C in darkness on PDA, colony superficial to gradually erumpent, with hyphae growing downward and fine, downy filaments immersed in medium, olivaceous.

##### Material examined.

China • Fujian Province, Taining County, on living leaves of *Camellia* sp. (26°54'22"N, 117°4'11"E), 3 September 2022, Yuwei Liu, TN-10 (IFRD 900-24, holotype), ex-type living culture IFRDCC 19-1198.

##### Notes.

In the phylogenetic tree, *Conidiocarpus tainingense* (IFRDCC 19-1198) clustered in a strongly supported lineage together with several previously unnamed *Conidiocarpus* strains reported by Abdollahzadeh et al. (2020), namely *Conidiocarpus* sp. (CBS 139818, CBS 139819, CBS 139820, CBS 139821, CPC 17778, CPC 20463, CPC 20465, CPC 20472, and CPC 21380), with BS/PP support values of 100%/1.00. Host/substrate metadata are available for several of these previously published strains. However, those strains were not re-examined morphologically in the present study, and therefore, direct strain-by-strain morphological comparison was not possible. The Chinese specimen IFRD 900-24/IFRDCC 19-1198 provides directly observed morphology, culture characteristics, and four-locus sequence data for this lineage. On this basis, the lineage is here formally named *Conidiocarpus tainingense*. Two additional unnamed strains reported by Abdollahzadeh et al. (2020), *Conidiocarpus* sp. (CPC 20464 and CPC 20468), were not included in this comparison because they were resolved in a separate subclade associated with *Conidiocarpus siamensis* (MFLUCC10-0061) rather than in the previously unnamed lineage corresponding to *C. tainingense*. Because *C. siamensis* (MFLUCC10-0061) is represented only by ITS and LSU in Abdollahzadeh et al. (2020), that subclade is not directly comparable at the same four-locus resolution used here for *C. tainingense*.

#### Conidiocarpus guilanensis

Taxon classification

Animalia

CapnodialesCapnodiaceae

Khodap., Mycological Progress 19 (2): 161 (2020).

9457C932-C227-56F3-98EB-1157F01593C5

Fig. 7

##### Description.

***Epiphytic*** on the leaf surface of *Rubus lambertianus*, forming a sooty coating on adaxial surface. Thallus composed of brown, septate, ellipsoidal, smooth-walled hyphae. ***Asexual morph. Conidiomata*** (384–578 × 25–41 µm, x̄ = 457 × 33 µm, *n* = 15) long, pycnidial, elongate, superficial, stipitate, with a ***long*-*stalk*** (173–378 µm, x̄ = 287 µm, *n* = 15), black, rigid, ***short neck*** (32–62 µm, x̄ = 50 µm, *n* = 15), and a distinctive ovoid-swollen conidiogenous region, the swollen area producing conidia inside. ***Conidiomata apex*** gradually light brown to hyaline, with a circular ***ostiole*** (7–18 µm, x̄ = 12 µm, *n* = 15), consisting of rectangular, compact cells, surrounded by hyaline hyphae. ***Conidiogenous region*** (18–37 µm, x̄ = 25 µm, *n* = 15) wide, located in the upper-middle part of the conidiomata, brown, composed of cylindrical, thin-walled cells. ***Conidia*** (3.8–5 × 1.6–2.8 μm, x̄ = 4.4 × 2.3 μm, *n* = 30), hyaline, single-celled, ellipsoidal, smooth-walled, guttulate without distinct refractive oil droplet, exuding in creamy masses from the ostiole. ***Sexual morph***. Undetermined.

**Figure 7.**
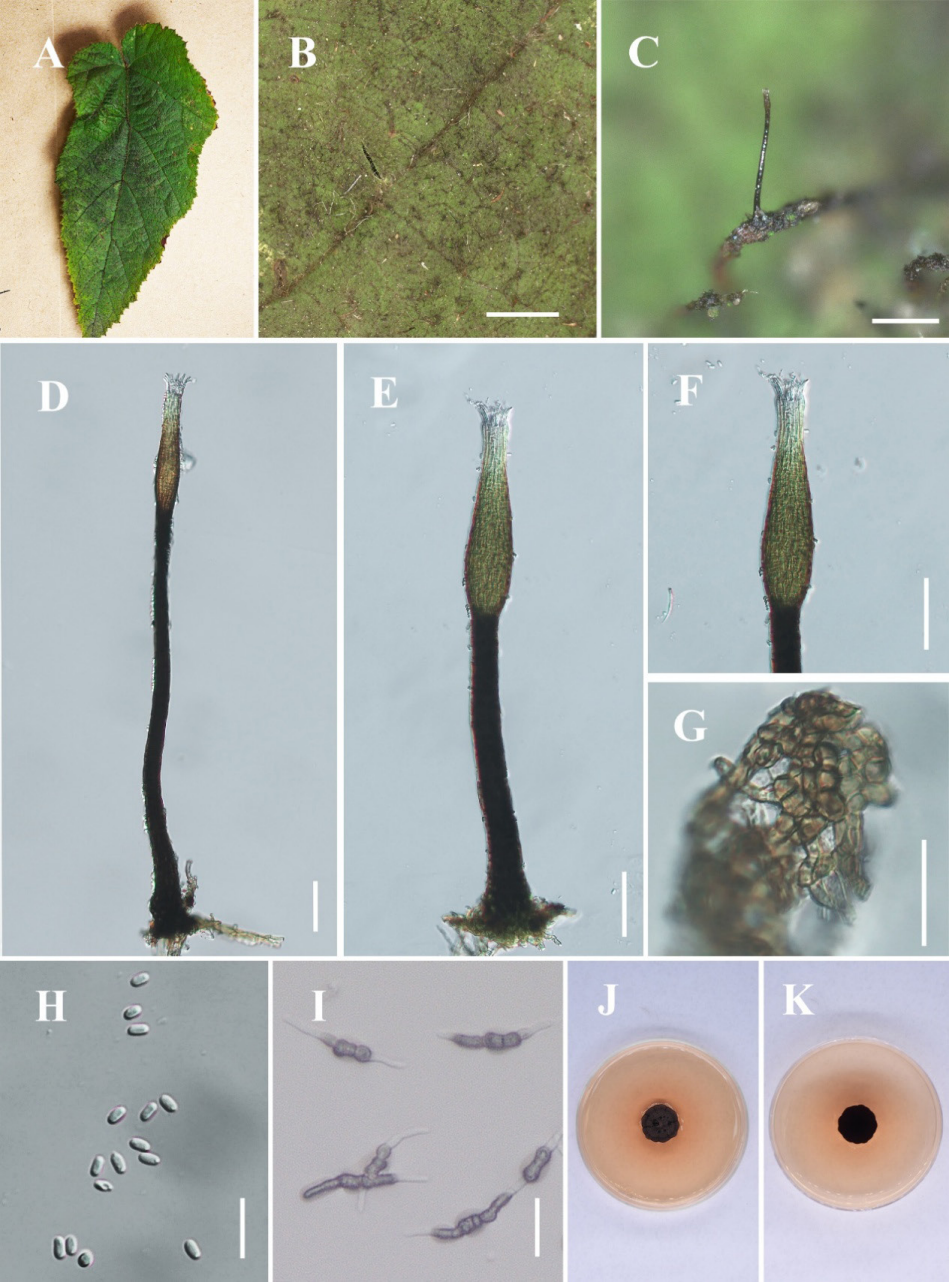
*Conidiocarpus guilanensis* (IFRDCC 25-0007). **A** Black mycelium covering the leaf surface; **B**, **C** Conidiomata on the host; **D, E** Conidioma; **F** Ostiole, neck, and a prominently ovoid-swollen conidiogenous region; **G** Mycelial network; **H** Conidia; **I** Germinated conidia; **J** Front view of the colony on PDA; **K** Reverse view of the colony on PDA. Scale bars: 2000 μm (**B**); 200 μm (**C**); 50 μm (**D–F**); 25 μm (**G–I**).

##### Culture characteristics.

Colonies slow growing, reaching 14.96 mm diam after 14 days at 26 °C in darkness on PDA, colony superficial to gradually erumpent, with hyphae growing downward and immersed in medium, olivaceous. The initially hyaline PDA medium is discolored to rust-colored as the fungal pigments diffuse into the substrate and accumulate over the cultivation period.

##### Material examined.

China • Guizhou Province, Zunyi City, on living leaves of *Rubus lambertianus* (28°02'24"N, 106°50'59"E), 23 October 2025, Jipeng Sun, ZY-15 (IFRD 900-14), living culture IFRDCC 25-0007.

##### Notes.

In the phylogenetic tree, two newly collected strains of *Conidiocarpus guilanensis* (IFRDCC 19-1213 and IFRDCC 25-0007), isolated from living leaves of *Ficus* sp. and *Rubus lambertianus*, respectively, clustered together with *C. guilanensis* (IRAN 2474C) in a single clade, with BS/PP support values of 100%/1.00. Because only ITS sequence data are available for *C. guilanensis* (IRAN 2474C), and no LSU, *tef*1-α, or *rpb*2 sequences have been generated for this strain, molecular comparisons could be conducted only for the ITS region. Sequence comparison revealed that *C. guilanensis* (IFRDCC 25-0007) shows no nucleotide differences in the ITS region compared with *C. guilanensis* (IRAN 2474C), and no significant morphological differences were observed between these two strains (Khodaparast et al. 2020).

When compared with *C. guilanensis* (IFRDCC 19-1213), *C. guilanensis* (IFRDCC 25-0007) exhibits a more distinct neck and a clearly ovoid swollen conidiogenous region. However, these morphological differences are minor and are not accompanied by phylogenetic separation; they are therefore interpreted here as intraspecific variation rather than evidence for species-level separation. The morphological features of *C. guilanensis* (IFRDCC 19-1213) are illustrated in Suppl. material 2. The two Chinese strains represent the first record of *C. guilanensis* from China. In addition, complete four-locus sequence data, including ITS, LSU, *tef*1-α, and *rpb*2, are provided for *C. guilanensis* (IFRDCC 25-0007).

#### Hyphopolychaeton

Taxon classification

Animalia

Capnodiaceae

J.P. Sun & X.Y. Zeng gen. nov.

EF70B0B3-B29B-5A8F-AFC9-B85223C3095E

Index Fungorum: IF904325

##### Type species.

*Hyphopolychaeton duyunense* J.P. Sun & X.Y. Zeng, sp. nov.

##### Description.

Thallus ***epiphytic***, growing on sugary exudates produced by insects feeding on living leaves and branches. ***Asexual morph***. observed. Thallus superficial, dark brown, branched, septate, and constricted at the septa. Mycelium superficial, forming dark brown conidiomata. Conidiomata, pycnidial, subcylindrical, dark brown to black, with a long black basal stalk and an expanded apical part, lacking a distinct neck. Conidia ellipsoid to subcylindrical or subclavate, hyaline. ***Sexual morph***. not observed.

##### Notes.

This genus comprises three species: *Hyphopolychaeton duyunense*, *Hy. cengongense*, and *Hy. maolanense*. *Hyphopolychaeton* is distinguished from other genera in *Capnodiaceae* by the ellipsoidal swollen region of the conidiogenous structure, which is located at the apex of the conidiomata and lacks a well-developed neck.

#### Hyphopolychaeton duyunense

Taxon classification

Animalia

Capnodiaceae

J.P. Sun & X.Y. Zeng sp. nov.

E614CC17-5CDB-52C4-8747-B8CC42CC2490

Index Fungorum: IF905004

Fig. 8

##### Etymology.

This name refers to the location of “Duyun City,” where the complete specimens were collected.

**Figure 8.**
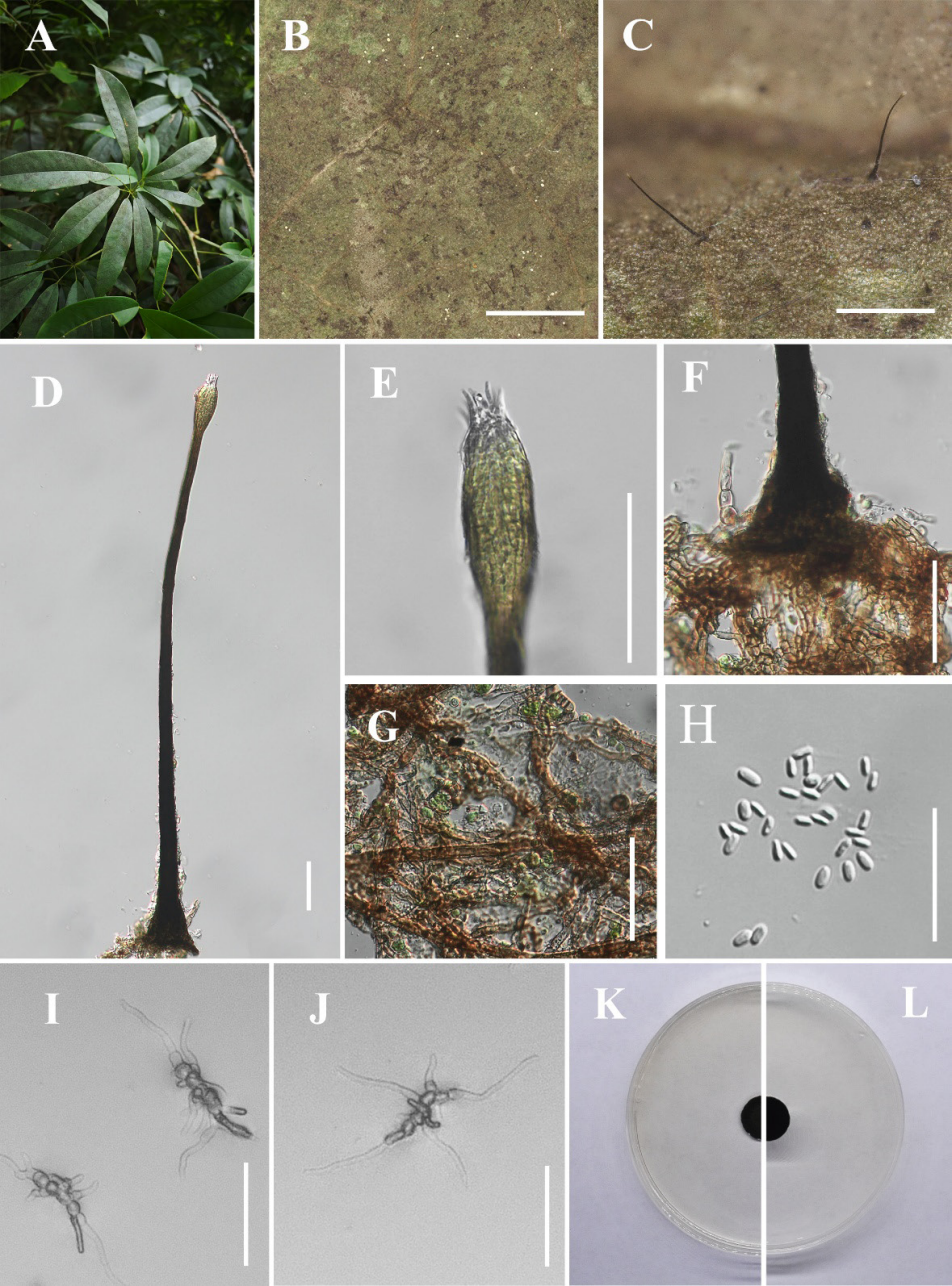
*Hyphopolychaeton duyunense* (IFRD 99052, holotype). **A** Black mycelium covering the leaf surface; **B, C** Conidiomata on host; **D** Conidioma; **E** Ostiole; **F** Base of conidioma; **G** Mycelial network; **H** Conidia; **I, J** Germinated conidia; **K** Front view of the colony on PDA; **L** Reverse view of the colony on PDA. Scale bars: 1000 μm (**B**); 250 μm (**C**); 50 μm (**D, E**); 25 μm (**F, H**); 50 μm (**I, J**).

##### Diagnosis.

It differs from other species in the genus by having an ellipsoidal swollen part of the conidiogenous structure, which is located at the apex of the conidiomata and lacks a long neck.

##### Holotype.

IFRD 99052.

##### Description.

***Epiphytic*** on the leaf surface of *Laurus nobilis*, forming a sooty coating on adaxial surface. Thallus composed of brown, septate, ellipsoidal, smooth-walled hyphae. ***Asexual morph. Conidiomata*** (612–802 × 27–62 µm, x̄ = 677 × 47 µm, *n* = 20) long, pycnidial, elongate, superficial, stipitate, with a ***long*-*stalk*** (357–611 µm, x̄ = 528 µm, *n* = 20), black, rigid, ***lacking neck***, and a distinctive ovoid-swollen conidiogenous region, the swollen area producing conidia inside. ***Conidiomata apex*** gradually light brown to hyaline, with a circular ***ostiole*** (10–17 µm, x̄ = 13 µm, *n* = 20), consisting of rectangular, compact cells, surrounded by hyaline hyphae. ***Conidiogenous region*** (16–26 μm, x̄ = 20 µm, *n* = 20) wide, located in the apex part of the conidiomata, brown, composed of cylindrical, thin-walled cells. ***Conidia*** (3.4–4.9 × 1.7–2.8 μm, x̄ = 4.1 × 2.1 μm, *n* = 30), hyaline, single-celled, ellipsoidal, smooth-walled, guttulate without distinct refractive oil droplet, exuding in creamy masses from the ostiole. ***Sexual morph***. Undetermined.

##### Culture characteristics.

Colony up to 8.4 mm diam at 26 °C on PDA after 14 days in darkness. Colony superficial to erumpent, velvety, with an entire margin, margin black, center olive green.

##### Material examined.

China • Guizhou Province, Duyun City, on living leaves of *Laurus nobilis*. (26°22'03"N, 107°22'43"E), 30 May 2025, Jipeng Sun, Du18-1 (IFRD 99052, holotype), ex-type living culture IFRDCC 25-0011.

##### Notes.

In the phylogenetic tree, *Hyphopolychaeton duyunense* is represented by nine strains from eight specimens (IFRDCC 25-0011, IFRDCC 25-0013, IFRDCC 25-0014, IFRDCC 25-0008, IFRDCC 25-0015, IFRDCC 25-0010, IFRDCC 25-0009, IFRDCC 25-0012, and IFRDCC 25-0016). These strains formed a distinct lineage with strong support (BS/PP = 99%/1.00) and were recovered as a lineage closely related to *Conidiocarpus* and *Phragmocapnias*. *Hyphopolychaeton duyunense* is most closely related to *Hy. cengongense*. Pairwise sequence comparisons between *Hy. duyunense* (IFRDCC 25-0011) and *Hy. cengongense* (IFRDCC 25-0020) showed no nucleotide differences in ITS, LSU, and *tef*1-α, whereas the *rpb*2 region showed 99.19% similarity (859/866 bp, 1 gap). In the combined four-locus phylogeny, however, strains of *Hy. duyunense* and *Hy. cengongense* were resolved as two distinct clades, with no intermixing between the two lineages. Additional pairwise comparisons showed that the lowest *rpb*2 similarity within *Hy. duyunense* was 99.24% (920/927 bp, 2 gaps), and that within *Hy. cengongense* was 99.88% (864/865 bp, 0 gaps). Thus, the *rpb*2 divergence between the two species is slightly greater than the maximum intraspecific divergence observed within either species in the present sampling. Nevertheless, the recognition of these two species is not based on *rpb*2 divergence alone. Morphologically, *Hy. duyunense* differs markedly from *Hy. cengongense* in having much larger conidiomata (612–802 μm vs. 132–188 μm), much longer stalks (357–611 μm vs. 13–33 μm), narrower ostioles (10–17 μm vs. 16–37 μm), and narrower conidiogenous structures (16–26 μm vs. 23–52 μm) (this study, below). These differences are substantial and non-overlapping and are therefore interpreted, together with the independent four-locus phylogenetic lineages, as stable interspecific distinctions rather than simple intraspecific variation.

#### Hyphopolychaeton cengongense

Taxon classification

Animalia

Capnodiaceae

J.P. Sun & X.Y. Zeng sp. nov.

F6E07C87-FAE8-5341-997D-809072B21FEF

Index Fungorum: IF905005

Fig. 9

##### Etymology.

This name refers to the location of “Cengong County,” where the complete specimens were collected.

**Figure 9.**
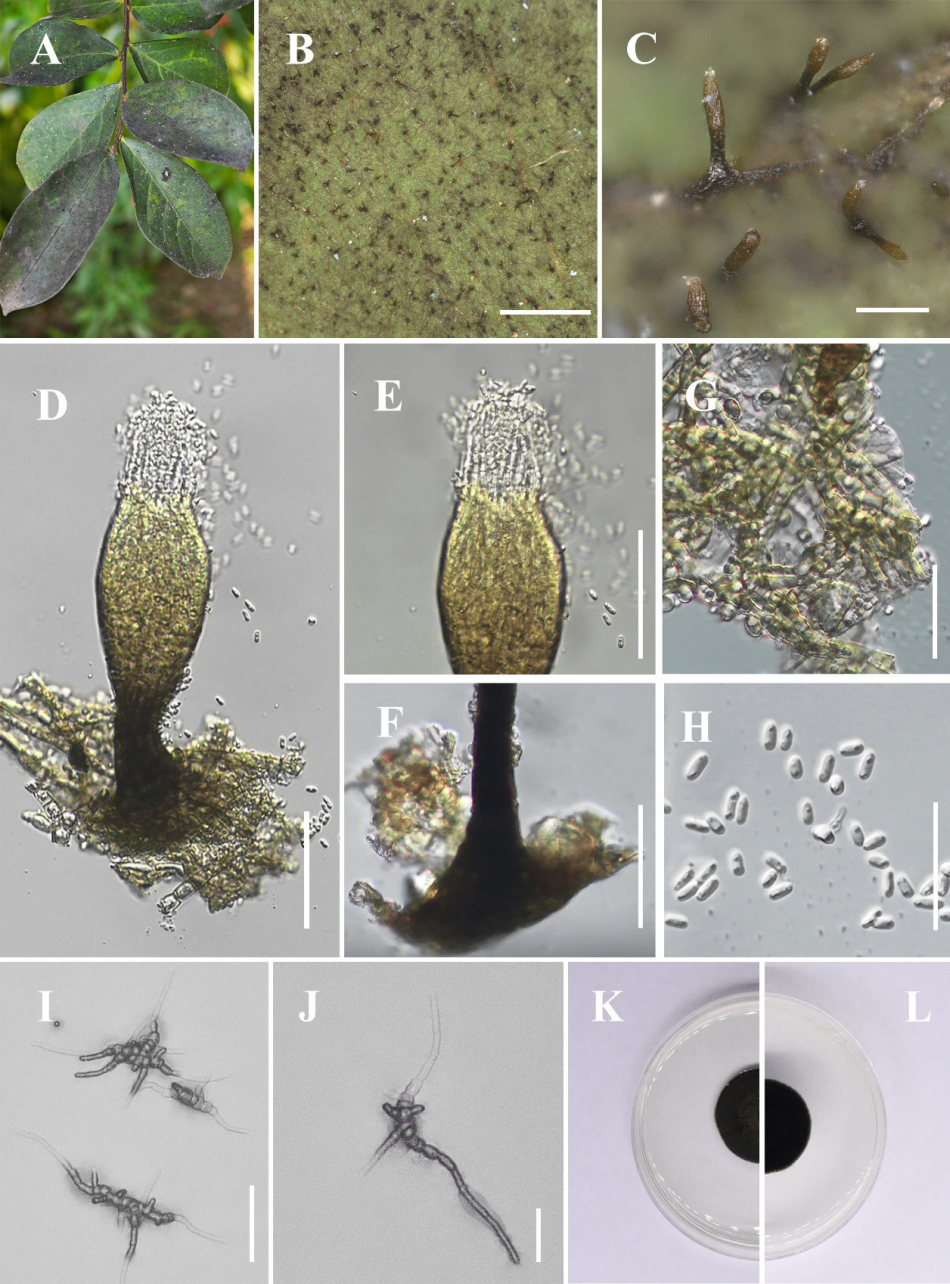
*Hyphopolychaeton cengongense* (IFRD 900-23, holotype). **A** Black mycelium covering the leaf surface; **B, C** Conidiomata on host; **D** Conidioma; **E** Ostiole; **F** Base of conidioma; **G** Mycelial network; **H** Conidia; **I, J** Germinated conidia; **K** Front view of the colony on PDA; **L** Reverse view of the colony on PDA. Scale bars: 500 μm (**B**); 50 μm (**C–H**); 25 μm (**I, J**).

##### Diagnosis.

Differs from *Hyphopolychaeton duyunense* by significantly smaller conidiomata, wider ostioles, and wider conidiogenous structure.

##### Holotype.

IFRD 900-23.

##### Description.

***Epiphytic*** on the leaf surface of *Lagerstroemia indica*, forming a sooty coating on adaxial surface. Thallus composed of brown, septate, ellipsoidal, smooth-walled hyphae. ***Asexual morph. Conidiomata*** (132–188 × 13–33 µm, x̄ = 156 × 32 µm, *n* = 20) long, pycnidial, elongate, superficial, stipitate, with a ***short*-*stalk*** (27–64 μm, x̄ = 48 µm, *n* = 20), black, rigid, ***lacking neck***, and a distinctive ovoid-swollen conidiogenous region, the swollen area producing conidia inside. ***Conidiomata apex*** gradually light brown to hyaline, with a circular ***ostiole*** (16–37 µm, x̄ = 24 µm, *n* = 20), consisting of rectangular, compact cells, surrounded by hyaline hyphae. ***Conidiogenous region*** (23–52 μm, x̄ = 35 µm, *n* = 20) wide, located in the apex part of the conidiomata, brown, composed of cylindrical, thin-walled cells. ***Conidia*** (3.6–6.2 × 2–3 μm, x̄ = 5.2 × 2.4 μm, *n* = 30), hyaline, single-celled, ellipsoidal, smooth-walled, guttulate with 1–2 distinct refractive oil droplets, exuding in creamy masses from the ostiole. ***Sexual morph***. Undetermined.

##### Culture characteristics.

Colony up to 25.41 mm diam at 26 °C on PDA after 14 days in darkness. Colony superficial to erumpent, velvety, with an entire margin, margin black, center olive green.

##### Material examined.

China • Guizhou Province, Cengong County, on living leaves of *Lagerstroemia indica*. (27°11'55"N, 108°44'07"E), 14 July 2025, Jipeng Sun, Cg2-3 (IFRD 900-23, holotype), ex-type living culture IFRDCC 25-0020.

##### Notes.

In the phylogenetic tree, *Hyphopolychaeton cengongense* is represented by four strains (IFRDCC 25-0018, IFRDCC 25-0019, IFRDCC 25-0020, and IFRDCC 25-0017) from three specimens, which formed a distinct lineage with high ML bootstrap support (BS/PP = 97%/-). *Hy. cengongense* is most closely related to *Hy. duyunense*. Pairwise sequence comparisons between *Hy. cengongense* (IFRDCC 25-0020) and *Hy. duyunense* (IFRDCC 25-0011) showed no nucleotide differences in ITS, LSU, and *tef*1-α, whereas the *rpb*2 region showed 99.19% similarity (859/866 bp, 1 gap). Although this molecular difference is limited, the two taxa were resolved as separate clades in the combined four-locus phylogeny and differ in stable, non-overlapping morphological characters. Morphologically, *Hy. cengongense* differs from *Hy. duyunense* in having much smaller conidiomata (132–188 μm vs. 612–802 μm), much shorter stalks (13–33 μm vs. 357–611 μm), wider ostioles (16–37 μm vs. 10–17 μm), and wider conidiogenous structures (23–52 μm vs. 16–26 μm) (this study, above). Therefore, *Hy. cengongense* is treated as distinct from *Hy. duyunense* based on the combined evidence of phylogenetic separation, *rpb*2 differentiation, and stable morphology.

#### Hyphopolychaeton maolanense

Taxon classification

Animalia

Capnodiaceae

J.P. Sun & X.Y. Zeng sp. nov.

3561336D-9C8C-5F2E-8EA3-0B6C3C24B4BC

Index Fungorum: IF905006

Fig. 10

##### Etymology.

This name refers to the location of “Maolan County,” where the complete specimens were collected.

**Figure 10.**
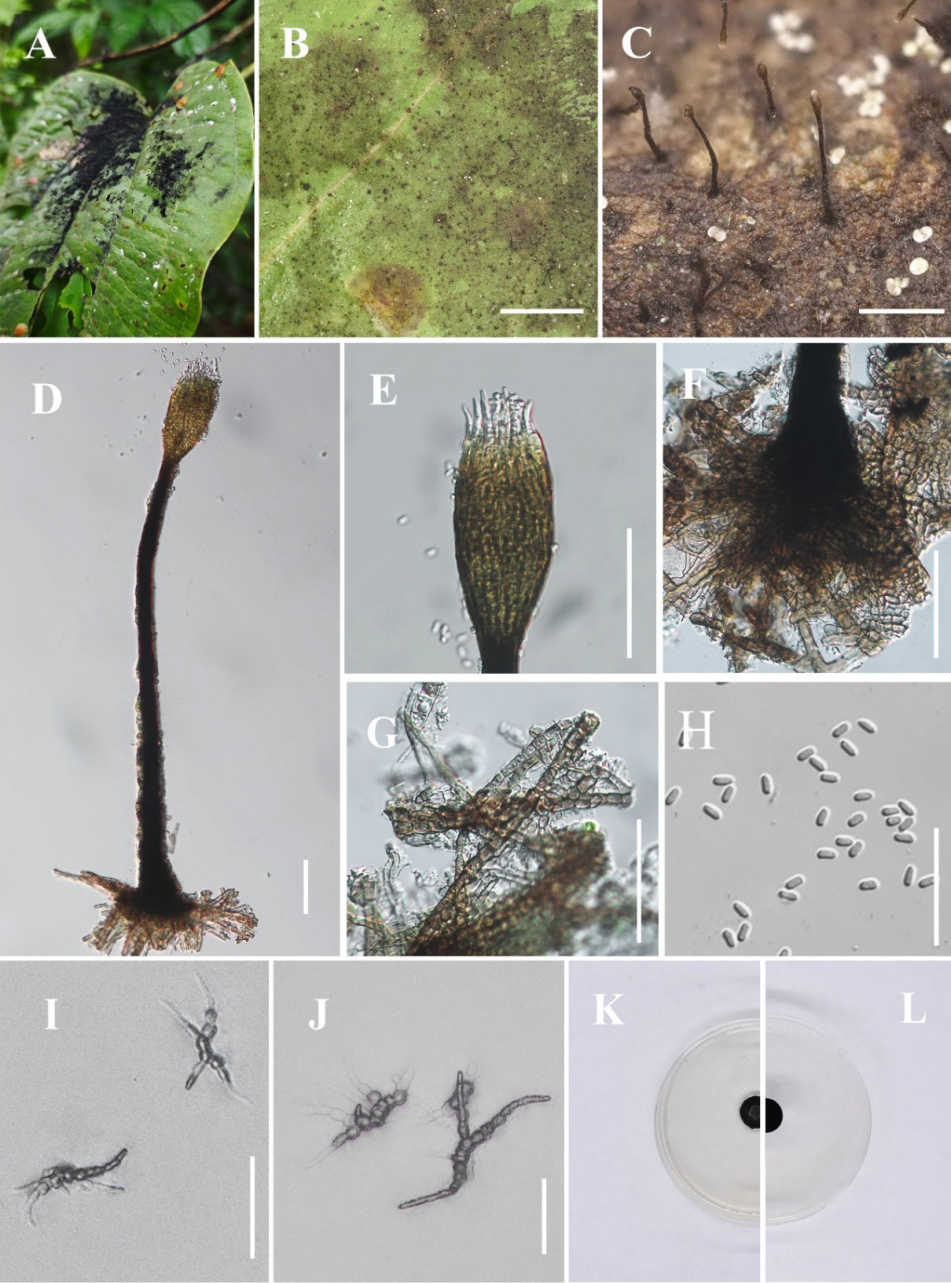
*Hyphopolychaeton maolanense* (IFRD 99053, holotype). **A** Black mycelium covering the leaf surface; **B, C** Conidiomata on host; **D** Conidioma; **E** Ostiole and a prominently ovoid-swollen conidiogenous region; **F** Base of conidioma; **G** Mycelial network; **H** Conidia; **I**, **J** Germinated conidia; **K** Front view of the colony on PDA; **L** Reverse view of the colony on PDA. Scale bars: 1000 μm (**B**); 250 μm (**C**); 50 μm (**D–G, I, J**); 25 μm (**H**).

##### Diagnosis.

Differs from *Hyphopolychaeton duyunense*, by slightly thick stalk, wider ostioles and conidiogenous structure. Differs from *Hyphopolychaeton cengongense*, by larger conidiomata and longer stalks.

##### Holotype.

IFRD 99053.

##### Description.

***Epiphytic*** on the leaf surface of *Smilax ocreata*, forming a sooty coating on adaxial surface. Thallus composed of brown, septate, ellipsoidal, smooth-walled hyphae. ***Asexual morph. Conidiomata*** (407–704 × 30–58 µm, x̄ = 523 × 45 µm, *n* = 20) long, pycnidial, elongate, superficial, stipitate, with a ***long*-*stalk*** (304–583 µm, x̄ = 420 µm, *n* = 20), black, rigid, ***lacking neck***, and a distinctive ovoid-swollen conidiogenous region, the swollen area producing conidia inside. ***Conidiomata apex*** gradually light brown to hyaline, with a circular ***ostiole*** (23–36 µm, x̄ = 29 µm, *n* = 20), consisting of rectangular, compact cells, surrounded by hyaline hyphae. ***Conidiogenous region*** (29–46 µm, x̄ = 38 µm, *n* = 20) wide, located in the apex part of the conidiomata, brown, composed of cylindrical, thin-walled cells. ***Conidia*** (3.4–5 × 1.5–3.4 μm, x̄ = 4.2 × 2.1 μm, *n* = 30), hyaline, single-celled, ellipsoidal, smooth-walled, guttulate without distinct refractive oil droplet, exuding in creamy masses from the ostiole. ***Sexual morph***. Undetermined.

##### Culture characteristics.

Colony up to 7.55 mm diam at 26 °C on PDA after 14 days in darkness. Colony superficial to erumpent, velvety, with an entire margin, margin black, center olive green.

##### Material examined.

China • Guizhou Province, Maolan County, on living leaves of *Smilax ocreata*. (27°17'26"N, 108°36'02"E), 14 July 2025, Jipeng Sun, M11 (IFRD 99053, holotype), ex-type living culture IFRDCC 25-0021.

##### Notes.

In the phylogenetic tree, *Hyphopolychaeton maolanense* (IFRDCC 25-0021) isolated in this study clustered in a well-supported clade together with *Hy. duyunense* and *Hy. cengongense*, forming a sister relationship with both species (BS/PP = 100%/1.00). *Hy. maolanense* (IFRDCC 25-0021) shares high sequence similarity with *Hy. duyunense* (IFRDCC 25-0011), showing 99.43% (520/523 bp, 0 gaps) similarity in the ITS region and 99.21% (881/888 bp, 3 gaps) in the LSU region; comparisons of the *tef*1-α and *rpb*2 regions revealed 99.19% (859/866 bp, 0 gaps) and 97.95% (906/925 bp, 0 gaps) similarity, respectively. Comparisons between *Hy. maolanense* (IFRDCC 25-0021) and *Hy. cengongense* (IFRDCC 25-0020) showed 99.59% (485/487 bp, 0 gaps) and 99.64% (828/831 bp, 0 gaps) similarity in the ITS and LSU regions, respectively, whereas the *tef*1-α and *rpb*2 regions exhibited 98.15% (795/810 bp, 0 gaps) and 97.92% (848/866 bp, 1 gap) similarity, respectively. Morphologically, *Hy. maolanense* (IFRDCC 25-0021) is distinguished from other species in the genus by possessing the second longest conidiomata, whereas *Hy. duyunense* (IFRDCC 25-0011) has the longest conidiomata and larger conidiogenous structures. Compared with *Hy. duyunense*, *Hy. maolanense* has smaller conidiomata (407–704 μm vs. 612–802 μm), wider ostioles (23–36 μm vs. 10–17 μm), and wider conidiogenous structures (29–46 μm vs. 16–26 μm) (this study, above). Compared with *Hy. cengongense* (IFRDCC 25-0020), *Hy. maolanense* has markedly larger conidiomata (407–704 μm vs. 132–188 μm) and substantially longer stalks (304–583 μm vs. 13–33 μm) (this study, above). The species was isolated from the host plant *Smilax ocreata*. Therefore, the strain is described here as a new species, *Hyphopolychaeton maolanense*.

#### Capnodium

Taxon classification

Animalia

CapnodialesCapnodiaceae

Mont., Ann. Sci. Nat. Bot. 11: 233 (1849).

5D489C5D-7A00-5CF3-968C-D622AF4D1F25

##### Type species.

*Capnodium citri* Berk. & Desm., in Berkeley, Journal of the Royal Horticultural Society 4: 252 (1849).

##### Notes.

The sooty mold genus *Capnodium* was established by Montagne and is assigned to the family *Capnodiaceae* (Hyde et al. 2013). Species of *Capnodium* are typically saprobic, developing on sugary exudates secreted by sap-feeding insects on the surfaces of living leaves. The thallus consists of superficial, irregular networks of gray-brown to brown mycelium, usually constricted at the septa (Chomnunti et al. 2011). Conidiomata are superficial and gregarious, often with a distinctly swollen basal to median region and a relatively short neck. Conidia are generally hyaline to pale brown, aseptate or occasionally septate, ellipsoidal, smooth-walled, and often guttulate. According to MycoBank (https://www.mycobank.org, accessed February 2026), *Capnodium* currently comprises approximately 151 species; however, molecular sequence data are available for only a small proportion of these taxa. This limited availability of sequence data has constrained robust phylogenetic inference and species delimitation within the genus. *Capnodium* can be distinguished from other genera in *Capnodiaceae* primarily by the position of the swollen region of the conidiomatal structure, which is typically located in the median to basal part of the conidioma, forming a conspicuous swollen base. This diagnostic character remains one of the most reliable morphological features for generic delimitation within the family.

#### Capnodium neocoffeicola

Taxon classification

Animalia

CapnodialesCapnodiaceae

Abdollahz. & Crous, Stud. Mycol. 95: 396 (2020)

A878B1B1-678E-57F9-B3A6-3B08DDE81C73

Fig. 11

##### Description.

***Epiphytic*** on the leaf surface of *Ficus* sp., forming a sooty coating on adaxial surface. Thallus composed of brown, septate, ellipsoidal, smooth-walled hyphae. ***Asexual morph. Conidiomata*** (158–292 μm, x̄ = 254 μm, *n* = 10) long, pycnidial, elongate, superficial, stipitate, ***sessile or with short stalk*** (29–48 × 25–37 μm, x̄ = 40 × 27 μm, *n* = 10), black, rigid, ***long neck*** (45–137 μm, x̄ = 111 μm, *n* = 10), and a distinctive ovoid-swollen conidiogenous region located at the lower middle part of the conidiomata, the swollen area producing conidia inside. ***Conidiomata apex*** gradually light brown to hyaline, with a circular ***ostiole*** (7–10 µm, x̄ = 9 µm, *n* = 10), consisting of rectangular, compact cells, surrounded by hyaline hyphae. ***Conidiogenous region*** (23–37 µm, x̄ = 31 µm, *n* = 10) wide, brown, composed of cylindrical, thin-walled cells. ***Conidia*** (3.5–5.2 × 1.6–2.3 μm, x̄ = 4.2 × 1.9 μm, *n* = 30), hyaline, single-celled, ellipsoidal, smooth-walled, guttulate with 1–2 distinct refractive oil droplets, exuding in creamy masses from the ostiole. ***Sexual morph***. Undetermined.

**Figure 11.**
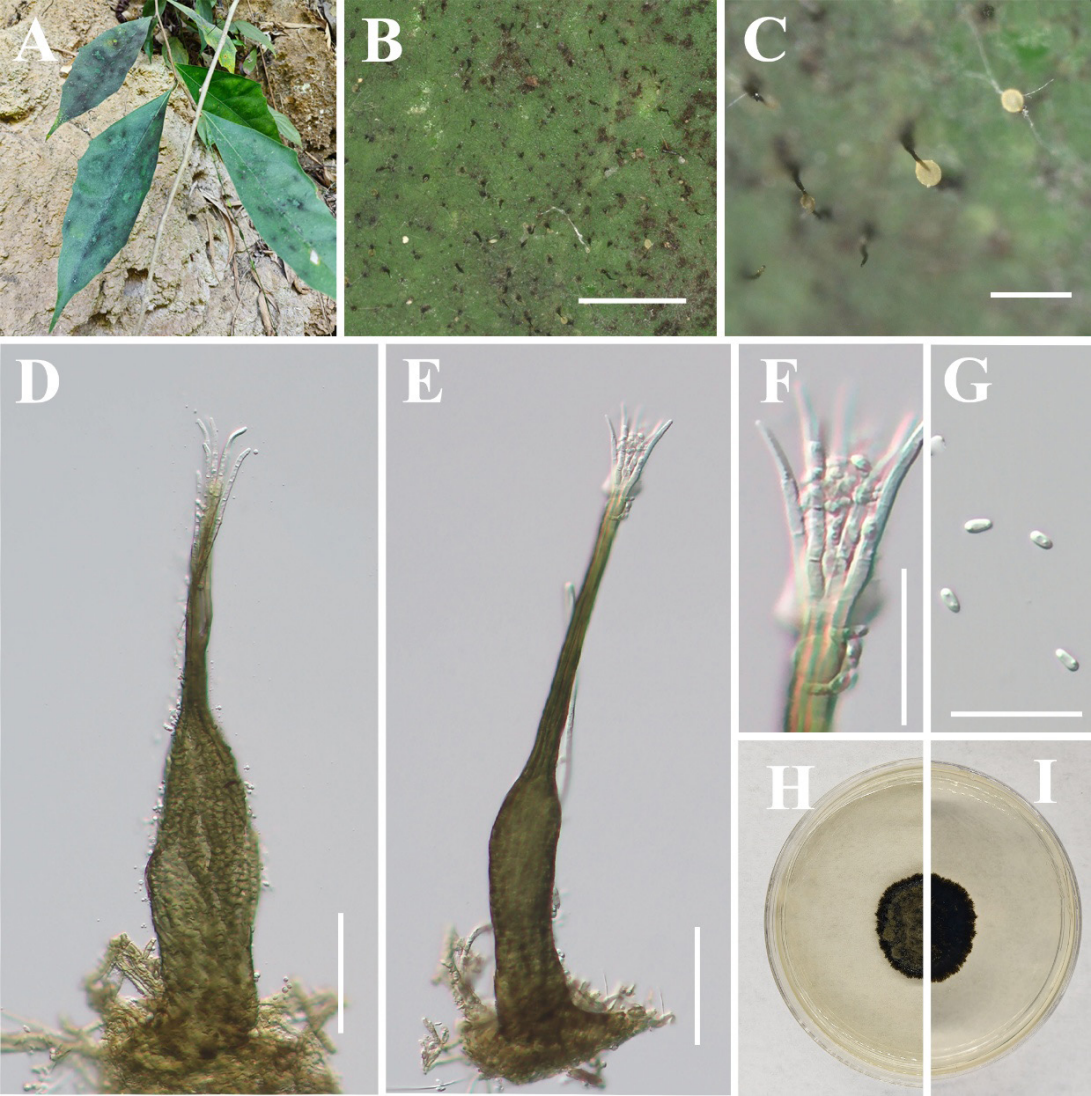
*Capnodium neocoffeicola* (IFRDCC 19-1201). **A** Black mycelium covering the leaf surface; **B, C** Conidiomata on host; **D**, **E** Conidioma; **F** Ostiole; **G** Conidia; **H** Front view of the colony on PDA; **I** Reverse view of the colony on PDA. Scale bars: 1000 μm (**B**), 200 μm (**C**), 50 μm (**D–F**), 20 μm (**G**).

##### Culture characteristics.

Colonies slow-growing, reaching 16.22 mm in diam after 14 days on PDA in darkness, colony superficial to erumpent, sometimes hyphae growing downwards and immersed into media. Margin black, center olive green, surface velvety with verrucose patches, hyphal branches evident at the margin.

##### Material examined.

China • Guizhou Province, Xingyi City, on living leaves of *Ficus* sp. (25°8'31"N, 104°57'12"E), 30 June 2023, Yuwei Liu, MLH5 (IFRD 99015), living culture IFRDCC 19-1201.

##### Notes.

In the phylogenetic tree, *Capnodium neocoffeicola* (IFRDCC 19-1201) and *Capnodium neocoffeicola* (CBS 139614) form a sister clade, with a BS/PP support value of 100%/0.99. The two strains exhibit no nucleotide differences in molecular data and show no significant differences in morphological characteristics (Abdollahzadeh et al. 2020). Therefore, the species represented by this strain is identified as the known species, *Capnodium neocoffeicola*. This species represents the first record in China, and it is also the first isolation from the plant host *Ficus* sp., with the acquisition of ITS, LSU, *tef*1-α, and *rpb*2 sequences.

#### Polychaeton

Taxon classification

Animalia

CapnodialesCapnodiaceae

(Pers.) Lév., In: Orbigny, Dict. Univ. Hist. Nat. 8: 493 (1846).

F75CBC79-C640-505D-B6C9-D91E8BC548A9

##### Type species.

*Polychaeton quercinum* (Pers.) Kuntze, Revisio generum plantarum 3 (3) (1891).

##### Notes.

*Polychaeton* was originally established by Persoon (1822) as a subgenus within *Fumago* and was later elevated to generic rank by Léveillé (1847). The taxonomic concept of *Polychaeton* has subsequently been re-evaluated by Hughes (1976) and Chomnunti et al. (2011). Hughes (1976) recognized *Polychaeton citri* and *P. quercinum* as representative species of the genus and formally designated *P. quercinum* as the lectotype. In contrast, Chomnunti et al. (2011) interpreted *Capnodium* as the sexual morph associated with *Polychaeton* and, in accordance with the “one fungus = one name” principle, adopted *Capnodium* as the accepted genus. According to MycoBank (https://www.mycobank.org, April 2026), the genus *Polychaeton* currently comprises 20 species, although molecular sequence data are available for only a few species, including *P. citri* and *P. cengongense*. *Polychaeton* is distinguished from other genera by the position of the swollen conidiogenous region in the middle to lower part of the conidiomata and by the absence of a stalk (Chomnunti et al. 2011).

#### Polychaeton citruscola

Taxon classification

Animalia

CapnodialesCapnodiaceae

J.P. Sun & X.Y. Zeng sp. nov.

8EC76C05-6996-5EA3-AB75-FE483312A9F7

Index Fungorum: IF904323

Fig. 12

##### Etymology.

The specific epithet “citruscola” is named after the “*Citrus*” genus to which the host plant of the fungus belongs, and the fungus was isolated from plants of this genus.

**Figure 12.**
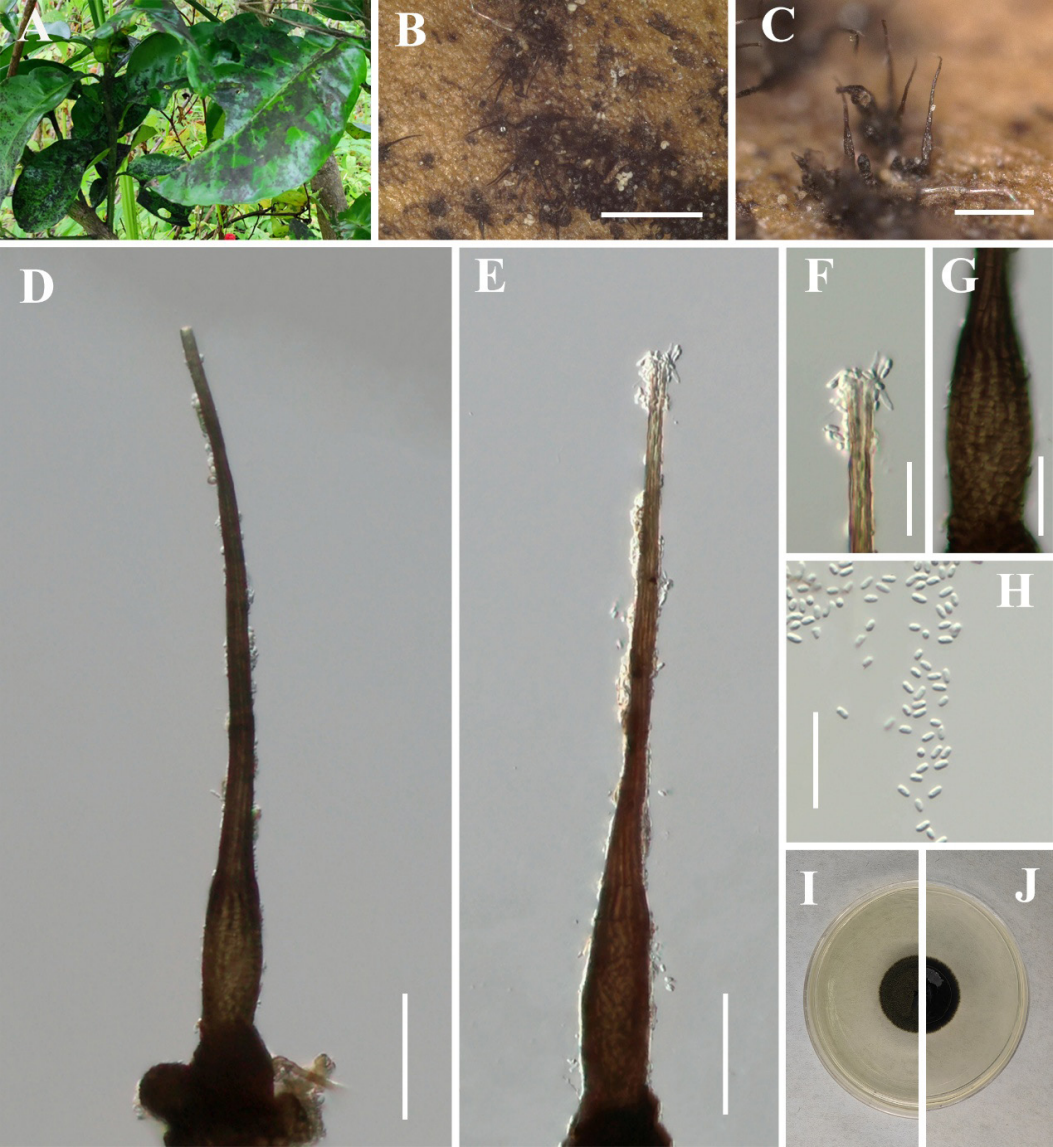
*Polychaeton citruscola* (IFRD 900-27, holotype). **A** Black mycelium covering the leaf surface; **B, C** Conidiomata on host; **D, E** Conidioma; **F** Ostiole and neck; **G** Prominently ovoid-swollen conidiogenous region; **H** Conidia; **I** Front view of the colony on PDA; **J** Reverse view of the colony on PDA. Scale bars: 500 μm (**B**), 200 μm (**C**), 50 μm (**D, E, G, H**), 20 μm (**F**).

##### Diagnosis.

Differs from *Polychaeton citri* (Pers.) Lév. 1846 by producing smaller conidiomata, narrower ostiole, and smaller conidia.

##### Holotype.

IFRD 900-27.

##### Description.

***Epiphytic*** on the leaf surface of *Citrus maxima*, forming a sooty coating on adaxial surface. Thallus composed of brown, septate, ellipsoidal, smooth-walled hyphae. ***Asexual morph. Conidiomata*** (204–340 µm, x̄ = 300 µm, *n* = 10) long, pycnidial, elongate, superficial, ***lack stalk and base***, with a ***long neck***, a prominently ovoid-swollen conidiogenous region, the swollen area producing conidia inside. ***Conidiomata apex*** gradually light brown to hyaline, with a circular ***ostiole*** (9.3–11 µm, x̄ = 10 µm, *n* = 10) diam, consisting of rectangular, compact cells, surrounded by hyaline hyphae. ***Conidiogenous region*** (26–32 µm, x̄ = 28 µm, *n* = 10) wide, located in the middle-lower part of the conidiomata, brown, composed of cylindrical, thin-walled cells. ***Conidia*** (3.1–4.8 × 1.8–2.5 µm, x̄ = 3.8 × 2.1 μm, *n* = 30), hyaline, single-celled, ellipsoidal, smooth-walled, guttulate without distinct refractive oil droplet, exuding in creamy masses from the ostiole. ***Sexual morph***. Undetermined.

##### Culture characteristics.

Colony up to 18.76 mm in diam on PDA at 26 °C after 14 days in darkness. Colony superficial, velvety, with an entire margin, olivaceous-green.

##### Material examined.

China • Guizhou Province, Jiangkou County, on living leaves of *Citrus maxima* (27°46'58"N, 108°44'17"E), 21 May 2022, Yuwei Liu, JK11 (IFRD 900-27, holotype), ex-type living culture IFRDCC 19-1216.

##### Notes.

In the phylogenetic tree, *Polychaeton citruscola* (IFRDCC 19-1216) forms a sister clade with *P. cengongense* (IFRDCC 25-0002, IFRDCC 25-0004) and *P. citri* (CBS 116435), with corresponding nodal support values of 100%/1.00 and 76%/1.00 (BS/PP), respectively. Only ITS, LSU, and *tef*1-α sequences were successfully obtained from *P. citruscola* (IFRDCC 19-1216), whereas repeated attempts to amplify the *rpb*2 gene were unsuccessful. This amplification failure is likely attributable to substantial sequence divergence or structural complexity in the *rpb*2 region, such as high GC content or the presence of insertions and deletions, which may interfere with primer annealing and PCR amplification efficiency. Sequence similarity analyses revealed that *P. citruscola* (IFRDCC 19-1216) shares 99.78% identity (464/465 bp, 0 gaps) in ITS, 99.23% (778/784 bp, 0 gaps) in LSU, and 98.26% (791/805 bp, 0 gaps) in *tef*1-α with *P. cengongense* (IFRDCC 25-0002). Comparisons with *P. citri* (CBS 116435) showed sequence similarities of 97.65% (457/468 bp, 3 gaps) in ITS, 99.17% (835/842 bp, 0 gaps) in LSU, and 93.72% (821/876 bp, 0 gaps) in *tef*1-α. Morphologically, *P. citruscola* (IFRDCC 19-1216) differs from *P. citri* (CBS 116435) in having smaller conidiomata (204–340 μm vs. 345–391 μm), narrower ostioles (9.3–11 μm vs. 13–15 μm), and smaller conidia (3.8–2.1 μm vs. 6.5–5 μm) (Chomnunti et al. 2011). Compared with *P. cengongense* (IFRDCC 25-0002), *P. citruscola* has narrower ostioles (9.3–11 μm vs. 10–23 μm) and narrower conidiogenous regions (26–32 μm vs. 30–62 μm) (Sun et al. 2026). *Polychaeton citri* was originally isolated from *Psidium guajava* and *Citrus* sp., whereas *P. citruscola* in the present study was obtained from *Citrus maxima* in China. Based on a combination of phylogenetic evidence, sequence divergence, morphological distinctions, and host association, the strain IFRDCC 19-1216 is described here as a new species, *Polychaeton citruscola*.

#### Leptoxyphium

Taxon classification

Animalia

CapnodialesCapnodiaceae

Speg., Physis, Rev. Soc. Arg. Cienc. Nat. 4 (17): 294 (1918).

0BB3FBA9-7CE7-514A-BDAE-C7F8EFE01694

##### Type species.

*Leptoxyphium graminum* (Pat.) Speg., Physis (Buenos Aires) 4 (17): 294 (1918).

##### Notes.

The sooty mold genus *Leptoxyphium* was introduced by Spegazzini (1918) and belongs to the family *Capnodiaceae* (Hyde et al. 2013). Species of *Leptoxyphium* are saprobic on sugary exudates produced by sap-feeding insects on the surfaces of living leaves. The thallus consists of superficial, irregular networks of mycelium that are gray-brown to brown and constricted at the septa (Chomnunti et al. 2011). Conidiomata are superficial and gregarious, sometimes helically twisted, with bulbous swollen bases and apices composed of cylindrical hyphae that expand into a funnel-shaped structure. Conidia are hyaline, aseptate, ellipsoidal, smooth-walled, and guttulate, with distinct refractive oil droplets. To date, only *Leptoxyphium kurandae* has been reported from China, collected on *Psidium guajava* in July 2012 (Yang et al. 2014). According to MycoBank (https://www.mycobank.org, accessed February 2026), the genus *Leptoxyphium* currently comprises 21 species, of which only six have available molecular sequence data, namely *L. cacuminum*, *L. citri*, *L. kurandae*, *L. madagascariense*, *L. glochidion*, and *L. fumago*. *Leptoxyphium* is distinguished from other genera in *Capnodiaceae* by a characteristic conidiomatal morphology, in which the swollen region of the conidiogenous structure is located at the apex of the conidiomata; this apical region is cup-shaped and serves as the site of conidium production.

#### Leptoxyphium fumago

Taxon classification

Animalia

CapnodialesCapnodiaceae

(Woron.) Crous, Fungal Syst. Evol. 6: 198 (2020)

D31DBB71-4D8B-58D3-9F4F-1887532D9159

Fig. 13

##### Description.

***Saprobic*** on sugary exudates from insects feeding on the surface of living leaves of *Maling bamboo*. Thallus superficial, branched, septate, pale brown, darkly pigmented at the septa, forming distinct constructional rings. ***Asexual morph***. Conidiomata, pycnidial, it originates from aggregated hyphae, with a bulbous base that consists of parallel hyphae and is straight to slightly flexuous. ***Conidiomata*** (273–378 μm, x̄ = 324 μm, *n* = 10) long, ***base*** (8–19 μm, x̄ = 15 μm, *n* = 10) wide, stalk light brown, conidiomata with cylindrical stalks expanding apically into a funnel-shaped cupula. ***Conidiogenous*** region resembling a ***cupula*** (45–61 × 30–47 μm, x̄ = 51 × 37 μm, *n* = 10), located in the cupulate apex of the conidiomata, brown, composed of cylindrical, thin-walled cells. ***Conidia*** (2.8–5.4 × 2.1–2.7 μm, x̄ = 3.9 × 2.4 μm, *n* = 30), hyaline, single-celled, ellipsoidal, smooth-walled, guttulate with 1–2 distinct refractive oil droplets, exuding in creamy masses from the ostiole. ***Sexual morph***. Undetermined.

**Figure 13.**
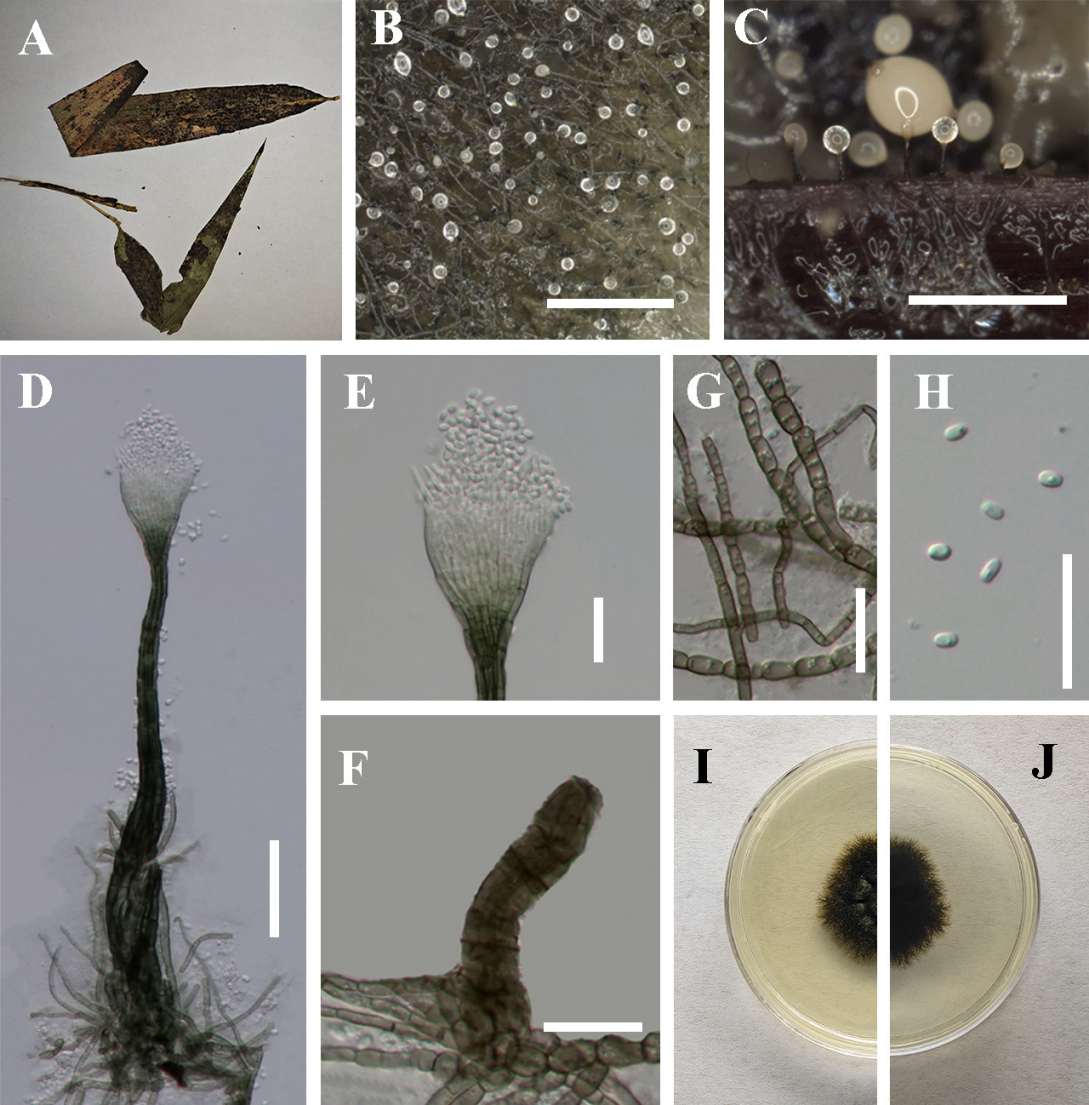
*Leptoxyphium fumago* (IFRDCC 19-1219). **A** Black mycelium covering the leaf surface; **B, C** Conidiomata on host; **D** Conidioma; **E** Cupulate ostiole; **F** Immature stages of conidioma; **G** Mycelial network; **H** Conidia; **I** Front view of the colony on PDA; **J** Reverse view of the colony on PDA. Scale bars: 1000 μm (**B**), 500 μm (**C**), 50 μm (**D**), 20 μm (**E–H**).

##### Culture characteristics.

Colonies black, loose, velvety, with sparse to moderate aerial mycelium and entire margins, reaching 23.91 mm in diam at 26 °C on PDA after 14 days in darkness.

##### Material examined.

China • Guizhou Province, Chishui City, on living leaves of *Maling bamboo* (28°2'1"N, 105°44'41"E), 12 April 2023, Yuwei Liu, CS1 (IFRD 99018), living culture IFRDCC 19-1219.

##### Notes.

In the phylogenetic analyses, *Leptoxyphium fumago* (IFRDCC 19-1219) was placed within the *L. fumago*-related lineage and occurred in close proximity to *L. fumago* (CBS 123.26), together with two unnamed *Leptoxyphium* strains, CBS 139618 and CPC 17767, with a BS/PP support value of 97%/1.00. An expanded genus-level phylogeny including all available named and unnamed *Leptoxyphium* strains from Abdollahzadeh et al. (2020) is provided in Suppl. material 3. This expanded sampling indicates that IFRDCC 19-1219 belongs to a broader *L. fumago*-related lineage rather than forming an exclusive two-strain pair with CBS 123.26. Multi-gene alignment between *L. fumago* (IFRDCC 19-1219) and *L. fumago* (CBS 123.26) revealed 100% similarity in ITS and LSU, *tef*1-α 99.66% (874/877 bp, 0 gaps), and *rpb*2 98.96% (1045/1056 bp, 0 gaps). These data support a close affinity between IFRDCC 19-1219 and CBS 123.26, but the presence of adjacent unnamed *Leptoxyphium* lineages in this part of the tree suggests that the species boundary around *L. fumago* remains incompletely resolved. Morphologically, the Chinese collection agrees with the general concept of *L. fumago* in having pycnidial conidiomata with a bulbous base, cylindrical stalks expanding apically into a funnel-shaped cupula, and hyaline, single-celled, ellipsoidal conidia. However, because directly comparable authenticated morphology for all closely related reference strains remains limited (Chomnunti et al. 2011; Yang et al. 2014; Abdollahzadeh et al. 2020; Crous et al. 2020; Lee and Choi 2024), IFRDCC 19-1219 is conservatively treated as a Chinese strain referable to *L. fumago*. The present collection provides detailed morphological documentation, culture characteristics, host information, and multilocus sequence data for a Chinese *L. fumago*-related strain from Maling bamboo, thereby expanding the reference dataset for this complex.

#### Leptoxyphium chishuiense

Taxon classification

Animalia

CapnodialesCapnodiaceae

J.P. Sun & X.Y. Zeng sp. nov.

44FED2D2-467F-5135-92F4-A1BC8E9B8815

Index Fungorum: IF904319

Fig. 14

##### Etymology.

This name refers to the location of “Chishui City,” where the complete specimens were collected.

**Figure 14.**
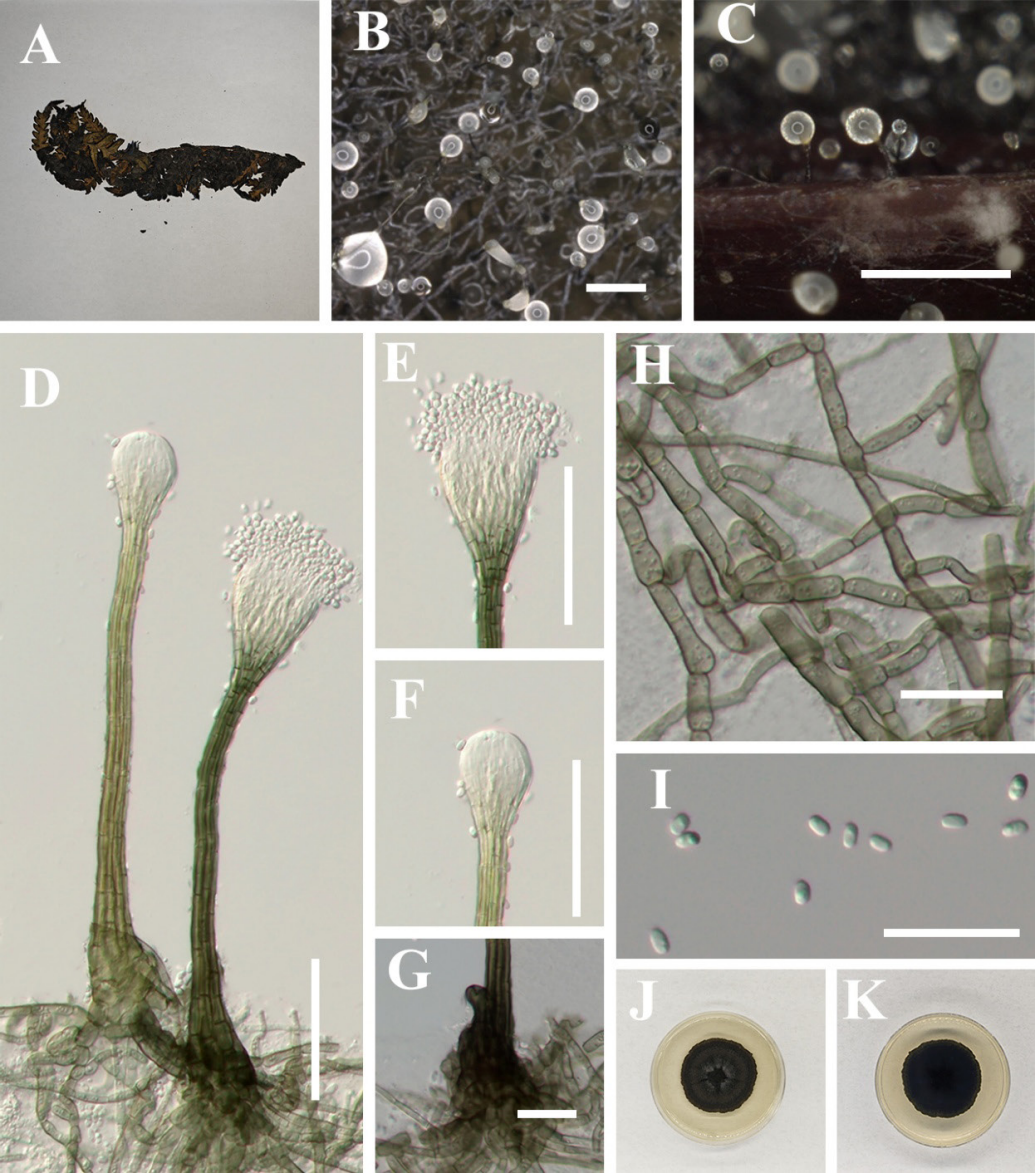
*Leptoxyphium chishuiense* (IFRD 900-28, holotype). **A** Black mycelium covering the leaf surface; **B, C** Conidiomata on host; **D** Conidioma; **E, F** Cupulate ostiole; **G** Base of conidioma; **H** Mycelial network; **I** Conidia; **J** Front view of the colony on PDA; **K** Reverse view of the colony on PDA. Scale bars: 250 μm (**B**), 500 μm (**C**), 50 μm (**D–F**), 20 μm (**G–I**).

##### Diagnosis.

Differs from *Leptoxyphium citri* Abdollahz. & Crous. by larger conidiomata and higher conidiogenous region. Differs from *Leptoxyphium madagascariense* Cheew. & Crous. by shorter conidiomata, narrower conidiogenous region and smaller conidia.

##### Holotype.

IFRD 900-28.

##### Description.

***Saprobic*** on sugary exudates from insects feeding on the surface of living leaves of *Alsophila* sp. Thallus superficial, branched, septate, pale brown, darkly pigmented at the septa, forming distinct constructional rings. ***Asexual morph***. Conidiomata pycnidial, it originates from aggregated hyphae, with a bulbous base that consists of parallel hyphae and is straight to slightly flexuous. ***Conidiomata*** (190–295 μm, x̄ = 241 μm, *n* = 10) long, ***bases*** (5.6–16.6 μm, x̄ = 12.6 μm, *n* = 10) wide, long-stalk light brown, conidiomata with cylindrical long-stalks expanding apically into a funnel-shaped cupula. ***Conidiogenous*** region resembling a ***cupula*** (45–61 × 27–44 μm, x̄ = 51 × 34 μm, *n* = 10), located in the cupulate apex of the conidiomata, brown, composed of cylindrical, thin-walled cells. ***Conidia*** (3–5.6 × 2–2.6 μm, x̄ = 4.1 × 2.3 μm, *n* = 30), hyaline, single-celled, ellipsoidal, smooth-walled, guttulate with 1–2 distinct refractive oil droplets, exuding in creamy masses from the ostiole. ***Sexual morph***. Undetermined.

##### Culture characteristics.

Colonies black, loose, velvety, with sparse to moderate aerial mycelium and entire margins, reaching 39.53 mm in diam at 26 °C on PDA after 14 days in darkness.

##### Material examined.

China • Guizhou Province, Chishui City, on living leaves of *Alsophila* sp. (28°23'1"N, 105°44'41"E), 12 April 2023, Yuwei Liu, CS4 (IFRD 900-28, holotype), ex-type living culture IFRDCC 19-1222.

##### Notes.

In the phylogenetic tree, *Leptoxyphium chishuiense* is represented by two strains, IFRDCC 19-1222 and IFRDCC 19-1225, which form a well-supported lineage with a BS/PP support value of 98%/0.99. The species is closely related to *L. fumago*, *L. citri*, and *L. kurandae*. Molecular comparisons show that pairwise sequence comparisons between *L. chishuiense* (IFRDCC 19-1222) and *L. fumago* (CBS 123.26) showed 99.78% similarity in ITS (445/446 nucleotides, 1 gap), no nucleotide differences in LSU, 99.77% similarity in *tef*1-α (879/881 nucleotides, 0 gaps), and 98.95% similarity in *rpb*2 (946/956 nucleotides, 0 gaps). When compared with *L. citri* (CBS 451.66), sequence similarity was 99.75% in ITS (399/400 nucleotides, 1 gap), 99.34% in LSU (748/753 nucleotides, 0 gaps), 96.71% in *tef*1-α (852/881 nucleotides, 0 gaps), and 90.90% in *rpb*2 (869/956 nucleotides, 2 gaps). Two unnamed *Leptoxyphium* strains, CBS 135836 and CBS 139620, were recovered in close proximity to the *L. chishuiense* lineage, indicating the presence of closely related but still unresolved taxa in this sector of *Leptoxyphium*. Although morphology has been reported for modern isolates referable to *L. fumago*, detailed morphological documentation directly comparable to authenticated reference material remains limited, and original type material has not been available for direct comparison (Chomnunti et al. 2011; Yang et al. 2014; Abdollahzadeh et al. 2020; Crous et al. 2020; Lee and Choi 2024). Therefore, direct morphological comparison with *L. fumago* (CBS 123.26) was not possible. In the present study, *L. fumago* (IFRDCC 19-1219), treated as a Chinese strain referable to *L. fumago*, was used as an additional comparative strain. Compared with *L. fumago* (IFRDCC 19-1219), *L. chishuiense* has smaller conidiomata (190–295 μm vs. 273–378 μm) (this study, above). It differs from *L. citri* (CBS 451.66) in having larger conidiomata (190–295 μm vs. 53–153 μm) and larger conidiogenous regions (45–61 μm vs. 20–40 μm) (Abdollahzadeh et al. 2020). It differs from *L. madagascariense* (CBS 124766) in having smaller conidiomata (190–295 μm, x̄ = 241 μm vs. 200–300 μm, x̄ = 250 μm), narrower conidiogenous regions (x̄ = 34 μm vs. 35–60 μm), and smaller conidia (3–5.6 × 2–2.6 μm vs. 4.5–5 × 3–3.5 μm) (Abdel-Sater 2018; Cheewangkoon et al. 2009). Based on phylogenetic position, multilocus sequence divergence, and morphological differences, the two strains examined here are recognized as a distinct species, *Leptoxyphium chishuiense*.

## Discussion

The present study provides new morphological, ecological, and four-locus phylogenetic evidence for the diversity and taxonomy of *Capnodiaceae* in China. Based on field collections, morphological observations, culture characteristics, host information, and analyses of ITS, LSU, *tef*1-α, and *rpb*2 sequence data, several novel taxa and new records are recognized in *Conidiocarpus*, *Leptoxyphium*, *Polychaeton*, and the newly introduced genus *Hyphopolychaeton*. These results expand the confirmed diversity of *Capnodiaceae* in China and provide additional multilocus reference data for lineages that have previously been poorly represented in phylogenetic studies. They also provide an opportunity to reassess the taxonomic value of selected morphological characters within a multilocus phylogenetic framework.

The host and geographic information associated with the newly collected strains further indicates that *Capnodiaceae* diversity in China remains insufficiently documented. The collections examined here were obtained from several provinces in China, with sampling concentrated in Guizhou Province, and from a broad range of host plants and habitats. Several taxa were recovered from more than one host plant, including *Conidiocarpus cinnamomi*, *C. caucasicus*, *Hyphopolychaeton duyunense*, and *Hyphopolychaeton cengongense*. These patterns suggest that at least some *Capnodiaceae* taxa are not restricted to a single host species but may occur across different host plants within suitable subtropical habitats. The host plants and geographic sources of the newly collected taxa are summarized in Table 3.

At the same time, host association alone should not be treated as a primary criterion for species delimitation. The occurrence of the same species on different host plants, as observed in *C. cinnamomi* and *C. caucasicus*, supports a cautious interpretation of host-based differences. Conversely, host and geographic data are valuable when integrated with morphology and multilocus phylogeny because they document the ecological breadth and distribution of well-supported taxa. The new host and geographic records provided here therefore extend the known ecological and regional range of several *Capnodiaceae* species and provide a more reliable basis for comparisons of sooty mold diversity in China.

Previous studies have shown that generic and species boundaries in *Capnodiaceae* can be difficult to resolve because many taxa were described from limited morphological information or incomplete molecular datasets (Chomnunti et al. 2011; Abdollahzadeh et al. 2020). Ribosomal markers alone, particularly ITS and LSU, may provide insufficient resolution for closely related taxa, whereas the inclusion of protein-coding genes can improve phylogenetic discrimination (Abdollahzadeh et al. 2020). The present results are consistent with this view. In several *Conidiocarpus* lineages, ITS and LSU were highly conserved, whereas *tef*1-α and *rpb*2 provided additional signals for distinguishing closely related taxa. These findings support the use of combined morphological and multilocus molecular evidence for taxonomic decisions in *Capnodiaceae*. A similar pattern was observed in *Hyphopolychaeton*. Although *Hy. duyunense* and *Hy. cengongense* showed no nucleotide differences in ITS, LSU, and *tef*1-α, they were recovered as two distinct clades in the combined four-locus phylogeny. Their *rpb*2 divergence was slightly greater than the maximum intraspecific *rpb*2 divergence observed within either species in the present sampling, and this molecular differentiation was congruent with stable, non-overlapping morphological differences. This case illustrates that small locus-specific differences should not be interpreted in isolation but may provide useful supporting evidence when they are consistent with phylogenetic structure and diagnostic morphology.

Additional support for this interpretation is provided by the expanded *Leptoxyphium* phylogeny presented in Suppl. material 3, which includes all available unnamed *Leptoxyphium* strains. This supplementary analysis showed that several unnamed lineages occur close to described species within *Leptoxyphium*, indicating that species boundaries in this genus remain incompletely resolved. The placement of the Chinese *L. fumago* and *L. chishuiense* lineages was therefore interpreted conservatively in the context of this broader sampling. In particular, the expanded phylogeny helped clarify that close affinity to named reference strains should be evaluated together with adjacent unnamed lineages, rather than being interpreted solely from an exclusive sister-pair relationship in the main tree. This result further supports the assessment of *Leptoxyphium* species boundaries using expanded taxon sampling, multilocus sequence data, and detailed morphology.

Asexual morphology remains important for interpreting generic boundaries in the family. Although sexual morphs are known for some taxa, many available collections are represented mainly by asexual states, making conidiomatal structure and the position of the conidiogenous region especially informative. In the present study, the position of the swollen region within the conidioma was broadly congruent with major phylogenetic lineages. Species of *Conidiocarpus* typically possess a swollen conidiogenous region in the upper-middle part of the conidioma, whereas *Capnodium* has a median to lower swollen region, and *Leptoxyphium* has an apical cupulate region. The newly introduced genus *Hyphopolychaeton* is characterized by an apical ellipsoid conidiogenous region and the absence of a long neck. These observations indicate that the spatial position and structure of the conidiogenous region are useful diagnostic characters when interpreted together with multilocus phylogenetic evidence.

Minor quantitative morphological differences, however, should be interpreted cautiously. Thungdee et al. (2023) reported that the swollen regions of the conidiomata and the conidia of *Conidiocarpus siamensis* specimens were slightly narrower and smaller than those of the type material. Such variation suggests that small differences in the dimensions of conidiomata, swollen regions, ostioles, or conidia may represent intraspecific variation rather than species-level divergence. A similar approach was adopted here. In *C. caucasicus*, slight differences in conidiomatal and conidial dimensions were interpreted as intraspecific variation because they were not accompanied by clear molecular divergence. By contrast, species such as *C. luodianense*, *C. bijieense*, and *C. tainingense* were recognized not based on minor size differences alone but through the combined evidence of independent phylogenetic placement, informative divergence in protein-coding loci, and consistent morphological distinction from closely related taxa.

The establishment of *Hyphopolychaeton* further illustrates the value of integrating morphology with multilocus phylogeny. Phylogenetically, *Hyphopolychaeton* forms an independent lineage within *Capnodiaceae*. Morphologically, it differs from related genera in the position and structure of the conidiogenous region. Unlike *Conidiocarpus*, which generally has an upper-middle swollen region with a distinct neck, and *Leptoxyphium*, which has an apical cupulate region, *Hyphopolychaeton* has an apical ellipsoid conidiogenous region and lacks a long neck. The included species are also supported by the combined evidence of phylogenetic placement and stable morphological characters. The genus is therefore not proposed based on a single feature but on congruent morphological and molecular evidence.

Taken together, the present results show that morphology remains essential for taxonomy in *Capnodiaceae* but that its interpretation should be context-dependent. Minor size differences may reflect intraspecific, host-associated, or environmentally influenced variation, especially when molecular divergence is absent. Conversely, stable morphological differences become taxonomically informative when they correspond to independent phylogenetic lineages and divergence in informative loci. This combined approach provides a more reliable basis for distinguishing species-level divergence from morphological plasticity in sooty mold fungi.

Several historical taxa in *Capnodiaceae* still lack complete multilocus data or detailed morphological documentation. Nevertheless, the four-locus dataset generated here improves the phylogenetic resolution of several poorly known lineages and provides reference data for taxonomic reassessment. By combining morphology, culture characteristics, host information, geographic data, and multilocus sequence data from Chinese collections, this study strengthens the taxonomic framework of *Capnodiaceae* and contributes to a more natural classification of sooty mold fungi.

## Conclusion

This study provides a combined morphological and four-locus phylogenetic assessment of *Capnodiaceae* collections from China based on morphology, culture characteristics, host and geographic information, and four-locus phylogenetic analyses of ITS, LSU, *tef*1-α, and *rpb*2. The results support the recognition of one new genus, *Hyphopolychaeton*, and eight new species and document additional host and geographic records for several known taxa. These findings expand the confirmed diversity of *Capnodiaceae* in China and provide new multilocus reference data for lineages that were previously poorly represented.

The results also clarify the taxonomic value of selected morphological characters. The position and structure of the conidiogenous region within the conidioma were broadly congruent with major phylogenetic lineages and are useful for generic delimitation when interpreted together with molecular evidence. By contrast, minor quantitative differences in conidiomatal or conidial dimensions should be treated cautiously, especially when they are not accompanied by clear molecular divergence. Thus, the present study does not treat morphology as unreliable but shows that morphological characters are most informative when evaluated within a multilocus phylogenetic framework.

The host and geographic data summarized in this study further indicate that several *Capnodiaceae* taxa occur on multiple host plants in subtropical China. Host association was not used as a primary criterion for species delimitation, but it provides useful ecological and distributional context for well-supported taxa. Together with the genus-level diagnostic key, host/geographic summary, and the expanded *Leptoxyphium* phylogeny, the data generated here provide a strengthened reference framework for the taxonomy of *Capnodiaceae* and for future comparisons of sooty mold fungi.

## Supplementary Material

XML Treatment for Conidiocarpus

XML Treatment for Conidiocarpus cinnamomi

XML Treatment for Conidiocarpus luodianense

XML Treatment for Conidiocarpus bijieense

XML Treatment for Conidiocarpus caucasicus

XML Treatment for Conidiocarpus tainingense

XML Treatment for Conidiocarpus guilanensis

XML Treatment for Hyphopolychaeton

XML Treatment for Hyphopolychaeton duyunense

XML Treatment for Hyphopolychaeton cengongense

XML Treatment for Hyphopolychaeton maolanense

XML Treatment for Capnodium

XML Treatment for Capnodium neocoffeicola

XML Treatment for Polychaeton

XML Treatment for Polychaeton citruscola

XML Treatment for Leptoxyphium

XML Treatment for Leptoxyphium fumago

XML Treatment for Leptoxyphium chishuiense

## Additional information

### Artificial Intelligence (AI) use

The authors accept full responsibility for the content of the manuscript, including the disclosure of any use of AI.

No AI tools were used in the preparation of this manuscript.

### Adherence to national and international regulations

All the fungal strains used in this study have been legally obtained, respecting the Convention on Biological Diversity (Rio Convention).

### Author ORCIDs

J. Sun https://orcid.org/0009-0004-0065-6920

Y. Liu https://orcid.org/0000-0002-0346-149X

T. Chen https://orcid.org/0009-0004-7806-4503

E. Tarafder https://orcid.org/0000-0002-3680-3433

S. Hongsanan https://orcid.org/0000-0003-0550-3152

S. Chen https://orcid.org/0009-0005-4945-5094

C. Norphanphoun https://orcid.org/0000-0002-5756-7206

X. Deng https://orcid.org/0009-0003-3475-0096

Y. Feng https://orcid.org/0009-0000-8832-7355

S.C. Karunarathna https://orcid.org/0000-0001-7080-0781

X. Zeng https://orcid.org/0000-0003-1341-1004

### Data availability

All data supporting the findings of this study are available within the article and its supplementary materials. Newly generated sequences have been deposited in GenBank, and the accession numbers are provided in Table 2. The sequence alignment files used for phylogenetic analyses are available as supplementary materials. Suppl. material 1 provides host plant molecular identification information based on available partial *rbcL* sequence data; Suppl. material 2 illustrates the morphological features of *Conidiocarpus guilanensis*IFRDCC 19-1213; and Suppl. material 3 provides the expanded *Leptoxyphium* phylogeny, including all available named and unnamed *Leptoxyphium* strains.

## Supplementary materials

Supplementary material 1 Supplementary file 1 Data type zip https://binary.pensoft.net/file/1661997This dataset is made available under the Open Database License (http://opendatacommons.org/licenses/odbl/1.0/). The Open Database License (ODbL) is a license agreement intended to allow users to freely share, modify, and use this Dataset while maintaining this same freedom for others, provided that the original source and author(s) are credited. Jipeng Sun, Yuwei Liu, Tao Chen, Entaj Tarafder, Sinang Hongsanan, Shengxi Chen, Chada Norphanphoun, Xiaolu Deng, Yuzhe Feng, Tian Yang, Samantha C. Karunarathna, Xiangyu Zeng

Supplementary material 2 Supplementary file 2 Data type tif https://binary.pensoft.net/file/1661998This dataset is made available under the Open Database License (http://opendatacommons.org/licenses/odbl/1.0/). The Open Database License (ODbL) is a license agreement intended to allow users to freely share, modify, and use this Dataset while maintaining this same freedom for others, provided that the original source and author(s) are credited. Jipeng Sun, Yuwei Liu, Tao Chen, Entaj Tarafder, Sinang Hongsanan, Shengxi Chen, Chada Norphanphoun, Xiaolu Deng, Yuzhe Feng, Tian Yang, Samantha C. Karunarathna, Xiangyu Zeng

Supplementary material 3 Supplementary file 3 Data type pdf https://binary.pensoft.net/file/1661999This dataset is made available under the Open Database License (http://opendatacommons.org/licenses/odbl/1.0/). The Open Database License (ODbL) is a license agreement intended to allow users to freely share, modify, and use this Dataset while maintaining this same freedom for others, provided that the original source and author(s) are credited. Jipeng Sun, Yuwei Liu, Tao Chen, Entaj Tarafder, Sinang Hongsanan, Shengxi Chen, Chada Norphanphoun, Xiaolu Deng, Yuzhe Feng, Tian Yang, Samantha C. Karunarathna, Xiangyu Zeng

Supplementary material 4 Alignment file Data type zip https://binary.pensoft.net/file/1662000This dataset is made available under the Open Database License (http://opendatacommons.org/licenses/odbl/1.0/). The Open Database License (ODbL) is a license agreement intended to allow users to freely share, modify, and use this Dataset while maintaining this same freedom for others, provided that the original source and author(s) are credited. Jipeng Sun, Yuwei Liu, Tao Chen, Entaj Tarafder, Sinang Hongsanan, Shengxi Chen, Chada Norphanphoun, Xiaolu Deng, Yuzhe Feng, Tian Yang, Samantha C. Karunarathna, Xiangyu Zeng

## References

[B1] Abdel-Sater MA (2018) First record of *Leptoxyphium madagascariense* (*Dothideomycetes*, *Capnodiales*) from sugarcane juice. Plant Pathology & Quarantine 8: 15–22. 10.5943/ppq/8/1/3

[B2] Abdollahzadeh J, Groenewald JZ, Coetzee MPA et al. (2020) Evolution of lifestyles in *Capnodiales*. Studies in Mycology 95: 381–414. 10.1016/j.simyco.2020.02.004 PMC7426231 32855743

[B3] Ariyawansa HA, Hyde KD, Jayasiri SC et al. (2015) Fungal diversity notes 111–252-taxonomic and phylogenetic contributions to fungal taxa. Fungal Diversity 75: 27–274. 10.1007/s13225-015-0346-5

[B4] Bezerra JDP, Oliveira RJV, Paiva LM et al. (2017) Bezerromycetales and *Wiesneriomycetales* ord. nov. (class *Dothideomycetes*), with two novel genera to accommodate endophytic fungi from Brazilian cactus. Mycological Progress 16: 297–309. 10.1007/s11557-016-1254-0

[B5] Bose T, Reynolds DR, Berbee ML (2014) Common, unsightly and until now un-described: *Fumiglobus pieridicola* sp. nov., a sooty mold infesting *Pieris japonica* from western North America. Mycologia 106: 746–756. 10.3852/13-288 24891416

[B6] Capella-Gutiérrez S, Silla-Martínez JM, Gabaldón T (2009) trimAl: a tool for auto-mated alignment trimming in large-scale phylogenetic analyses. Bioinformatics 25: 1972–1973. 10.1093/bioinformatics/btp348 PMC2712344 19505945

[B7] Cheewangkoon R, Groenewald JZ, Summerell BA et al. (2009) Myrtaceae, a cache of fungal biodiversity. Persoonia 23: 55–85. 10.3767/003158509X474752 PMC2802731 20198162

[B8] Cheewangkoon R, Groenewald JZ, Hyde KD et al. (2012) Chocolate spot disease of Eucalyptus. Mycological Progress 11: 61–69. 10.1007/s11557-010-0728-8

[B9] Chomnunti P, Hongsanan S, Aguirre-Hudson B et al. (2014) The sooty moulds. Fungal Diversity 66: 1–36. 10.1007/s13225-014-0278-5

[B10] Chomnunti P, Schoch CL, Aguirre-Hudson B et al. (2011) *Capnodiaceae*. Fungal Diversity 51: 103–134. 10.1007/s13225-011-0145-6 PMC3377173 22737101

[B11] Crous PW, Groenewald JZ, Shivas RG et al. (2011) Fungal Planet descrip-tion sheets: 69–91. Persoonia 26: 108–156. 10.3767/003158511X581723 PMC3160798 22025808

[B12] Crous PW, Schoch CL, Hyde KD et al. (2009) Phylogenetic lineages in the *Capnodiales*. Studies in Mycology 64: 17–47. 10.3114/sim.2009.64.02 PMC2816965 20169022

[B13] Crous PW, Wingfield MJ, Schumacher RK et al. (2020) New and interesting fungi. 3. Fungal Systematics and Evolution 6: 157–231. 10.3114/fuse.2020.06.09 PMC7452156 32904192

[B14] Czachura P, Janik P, Piątek M (2025) *Chaetocapnodium magnum* and *Chaetocapnodium polonicum* from conifer resins disclose an unknown lifestyle in the *Capnodiales (Dothideomycetes)*. MycoKeys 119: 315–333. 10.3897/mycokeys.119.159094 PMC12280968 40697982

[B15] Hall TA (1999) BioEdit: A user-friendly biological sequence alignment editor and analysis program for Windows 95/98/NT. Nucleic Acids Symposium Series 41: 95–98.

[B16] Höhnel F (1910) Fragmenta zur Mykologie (Xi Mitteilung, Nr. 527 bis 573). Sitzungsberichte der Kaiserlichen Akademie der Wissenschaften in Wien, Abt. I, 119: 617–679. https://www.biodiversitylibrary.org/part/234030

[B17] Hongsanan S, Tian Q, Hyde KD et al. (2015) Two new species of sooty moulds, *Capnodium coffeicola* and *Conidiocarpus plumeriae* in *Capnodiaceae*. Mycosphere 6: 814–824. 10.5943/mycosphere/6/6/14

[B18] Hughes SJ (1976) Sooty Moulds. Mycologia 68: 693–820. 10.1080/00275514.1976.12019958

[B19] Hyde KD, Hongsanan S, Jeewon R et al. (2016) Fungal diversity notes 367–490: taxonomic and phylogenetic contributions to fungal taxa. Fungal Diversity 80: 1–270. 10.1007/s13225-016-0373-x

[B20] Hyde KD, Jones EBG, Liu JK et al. (2013) Families of *Dothideomycetes*. Fungal Diversity 63: 1–313. 10.1007/s13225-013-0263-4

[B21] Index Fungorum (2026) Index Fungorum. [accessed 1 January 2026] http://www.indexfungorum.org/Names/Names.asp

[B22] Jouraeva VA, Johnson DL, Hassett JP et al. (2006) Role of sooty mold fungi in accumulation of fine-particle-associated PAHs and metals on deciduous leaves. Environmental Research 102: 272–282. 10.1016/j.envres.2006.06.004 16890933

[B23] Katoh K, Standley DM (2013) MAFFT multiple sequence alignment software version 7: improvements in performance and usability. Molecular Biology and Evolution 30: 772–780. 10.1093/molbev/mst010 PMC3603318 23329690

[B24] Khodaparast SA (2006) A survey on *citrus* sooty mold fungi in Gilan Province, Iran. Rostaniha (Botanical Journal of Iran) 7: 59–65. https://civilica.com/doc/1542941

[B25] Khodaparast SA, Pourmoghaddam MJ, Amirmijani A et al. (2020) Phylogenetic structure of the Iranian *capnodiaceous* sooty mould fungi inferred from the se-quences of rDNA regions and TEF1-α. Mycological Progress 19: 155–169. 10.1007/s11557-019-01551-w

[B26] Kress WJ, Erickson DL, Jones FA et al. (2009) Plant DNA barcodes and a community phylogeny of a tropical forest dy-namics plot in Panama. Proceedings of the National Academy of Sciences 106: 18621–18626. 10.1073/pnas.0909820106 PMC2763884 19841276

[B27] Lee DJ, Choi YJ (2024) First report of *Leptoxyphium fumago* causing sooty mold on coffee (*Coffea arabica*) in Korea. Australasian Plant Pathology 53: 567–570. 10.1007/s13313-024-01011-4

[B28] Léveillé JH (1847) Mycologie, mycétologie. Dictionnaire Universel d’Histoire na-turelle. Victor Masson, Paris, 261–303.

[B29] Li WJ, McKenzie EHC, Liu JK et al. (2020) Taxonomy and phylogeny of hyaline-spored coelo-mycetes. Fungal Diversity 100: 279–801. 10.1007/s13225-020-00440-y

[B30] Liu YJ, Whelen S, Hall BD (1999) Phylogenetic relationships among ascomycetes: evidence from an RNA polymerase II subunit. Molecular Biology and Evolution 16: 1799–1808. 10.1093/oxfordjournals.molbev.a026092 10605121

[B31] Lu ZH, Wanasinghe DN, Madrid H et al. (2022) *Hyphocapnodia sichuanensis* gen. et sp. nov. (*Capnodiaceae*), a novel hyphomycete from Si-chuan Province, China. Phytotaxa 564: 084–094. 10.11646/phytotaxa.564.1.6

[B32] MycoBank (2026) MycoBank. [accessed 1 January 2026] https://www.mycobank.org

[B33] O’Donnell K, Sarver BAJ, Brandt ME et al. (2007) Phylogenetic diversity and microsphere array-based genotyping of human pathogenic *Fusaria*, including isolates from the multistate contact lens-associated U.S. keratitis outbreaks of 2005 and 2006. Journal of Clinical Microbiology 45: 2235–2248. 10.1128/JCM.00533-07 PMC1933018 17507522

[B34] Pem D, Hyde KD, McKenzie EHC et al. (2024) A comprehensive overview of genera in *Dothideomycetes*. My-cosphere 15: 2175–4568. 10.5943/mycosphere/15/1/18

[B35] Persoon CH (1822) Mycologia Europaea, seu Completa omnium fungorum in variis Europaeae regionibus detectorum enumeratio, methodo naturali disposita. Sectio prima. Erlangae: Impensis Ioannis Iacobi Palmii, 9–9.

[B36] Reeb V, Lutzoni F, Roux C (2004) Contribution of RPB2 to multilocus phylogenetic studies of the *Euascomycetes* (*Pezizomycotina*, Fungi) with special emphasis on the lichen-forming *Acarosporaceae* and evolution of polyspory. Molecular Phylogenetics and Evolution 32: 1036–1060. 10.1016/j.ympev.2004.04.012 15288074

[B37] Rehner SA, Samuels GJ (1994) Taxonomy and phylogeny of Gliocladium analysed from nuclear large subunit ribosomal DNA sequences. Mycological Research 98: 625–634. 10.1016/S0953-7562(09)80409-7

[B38] Reynolds DR, Gilbert GS (2005) Epifoliar fungi from Queensland, Australia. Austral-ian Systematic Botany 18: 265–289. 10.1071/SB04030

[B39] Reynolds DR (1998) *Limaciniaseta* gen. nov., a California sooty mould. Madroño 45: 250–254.

[B40] Ronquist F, Teslenko M, van der Mark P et al. (2012) MrBayes 3.2: Efficient Bayesian Phylo-genetic Inference and Model Choice Across a Large Model Space. Systematic Bi-ology 61: 539–542. 10.1093/sysbio/sys029 PMC3329765 22357727

[B41] Schoch CL, Crous PW, Groenewald JZ et al. (2009) A class-wide phylogenetic assessment of *Dothideomycetes*. Studies in Mycology 64: 1–15. 10.3114/sim.2009.64.01 PMC2816964 20169021

[B42] Spatafora JW, Sung G-H, Johnson D et al. (2006) A five-gene phylogeny of *Pezizomycotina*. Mycologia 98: 1018–1028. 10.1080/15572536.2006.11832630 17486977

[B43] Spegazzini C (1918) Notas Micológicas. Physis (Buenos Aires) 4: 281–295.

[B44] Sun JP, Tarafder E, Chen T et al. (2026) Two novel species of sooty mould fungi (*Capnodiales*, *Dothideomycetes*) from China. MycoKeys 129: 23–41. 10.3897/mycokeys.129.182290 PMC12946820 41766950

[B45] Tennakoon DS, Kuo C-H, Maharachchikumbura SSN et al. (2021) Taxonomic and phylogenetic contributions to *Celtis formosana*, *Ficus ampelas*, *F. septica*, *Macaranga tanarius* and *Morus australis* leaf litter inhabiting microfungi. Fungal Diversity 108: 1–215. 10.1007/s13225-021-00474-w

[B46] Theissen F (1915) Mykologische Abhandlungen. Verhandlungen der Kaiser-lich-Königlichen Zoologisch-Botanischen Gesellschaft in Wien 66: 231–234. https://www.biodiversitylibrary.org/item/238853

[B47] Thungdee S, Haituk S, Withee P et al. (2023) Unraveling *Capnodiaceae* species in Northern Thailand. Phy-totaxa 620: 143–156. 10.11646/phytotaxa.620.2.2

[B48] Tian WH, Liu JW, Jin Y et al. (2024) Morphological and phylogenetic studies of *Ascomycota* from gymno-sperms in Sichuan Province, China. Mycosphere 15: 1794–1900. 10.5943/mycosphere/15/1/16

[B49] Vilgalys R, Hester M (1990) Rapid genetic identification and mapping of enzymati-cally amplified ribosomal DNA from several *Cryptococcus* species. Journal of Bacteriology 172: 4238–4246. 10.1128/jb.172.8.4238-4246.1990 PMC213247 2376561

[B50] White TJ, Bruns T, Lee S et al. (1990) Amplification and direct sequencing of fungal ribosomal RNA genes for phylogenetics. PCR Protocols 1990: 315–322. 10.1016/B978-0-12-372180-8.50042-1

[B51] Wijayawardene NN, Hyde KD, Dai DQ et al. (2022) Outline of Fungi and fungus-like taxa–2021. Mycosphere 13: 53–453. 10.5943/mycosphere/13/1/2

[B52] Wijayawardene NN, Crous PW, Kirk PM et al. (2014) Naming and outline of *Dothideomycetes*–2014 including proposals for the protection or suppression of generic names. Fungal Diversity 69: 1–55. 10.1007/s13225-014-0309-2 PMC4896388 27284275

[B53] Xie JT, Liu X, Qin ZL et al. (2024) Evolution and related pathogenic genes of *Pseudodiploospora longispora* on *Morchella* based on genomic characterization and comparative genomic analysis. Scientific Reports 14: 18588. 10.1038/s41598-024-69421-4 PMC11316761 39127740

[B54] Yang CL, Li XY, Xiang SS et al. (2025) Microfungi associ-ated with plant diseases on horticultural vegetation in southwestern China. Myco-sphere 16: 1861–2001. 10.5943/mycosphere/16/1/11

[B55] Yang H, Ariyawansa HA, Wu HX et al. (2014) The genus *Leptoxyphium (Capnodiaceae)* from China. Phytotaxa 176: 174–183. 10.11646/phytotaxa.176.1.17

[B56] Zeng XY, Tan TJ, Tian FH et al. (2023) OFPT: a one-stop software for fungal phylogeny. Mycosphere 14: 1730–1741. 10.5943/mycosphere/14/1/20

