# Supplementary figures and images for: Morphology and multilocus phylogeny illuminate novel taxa in Capnodiaceae (Ascomycota, Dothideomycetes, Capnodiales)
Source: IMA Fungus. 2026 May 29;17:e188051. doi: 10.3897/imafungus.17.188051 (PMC13241920; doi:10.3897/imafungus.17.188051)

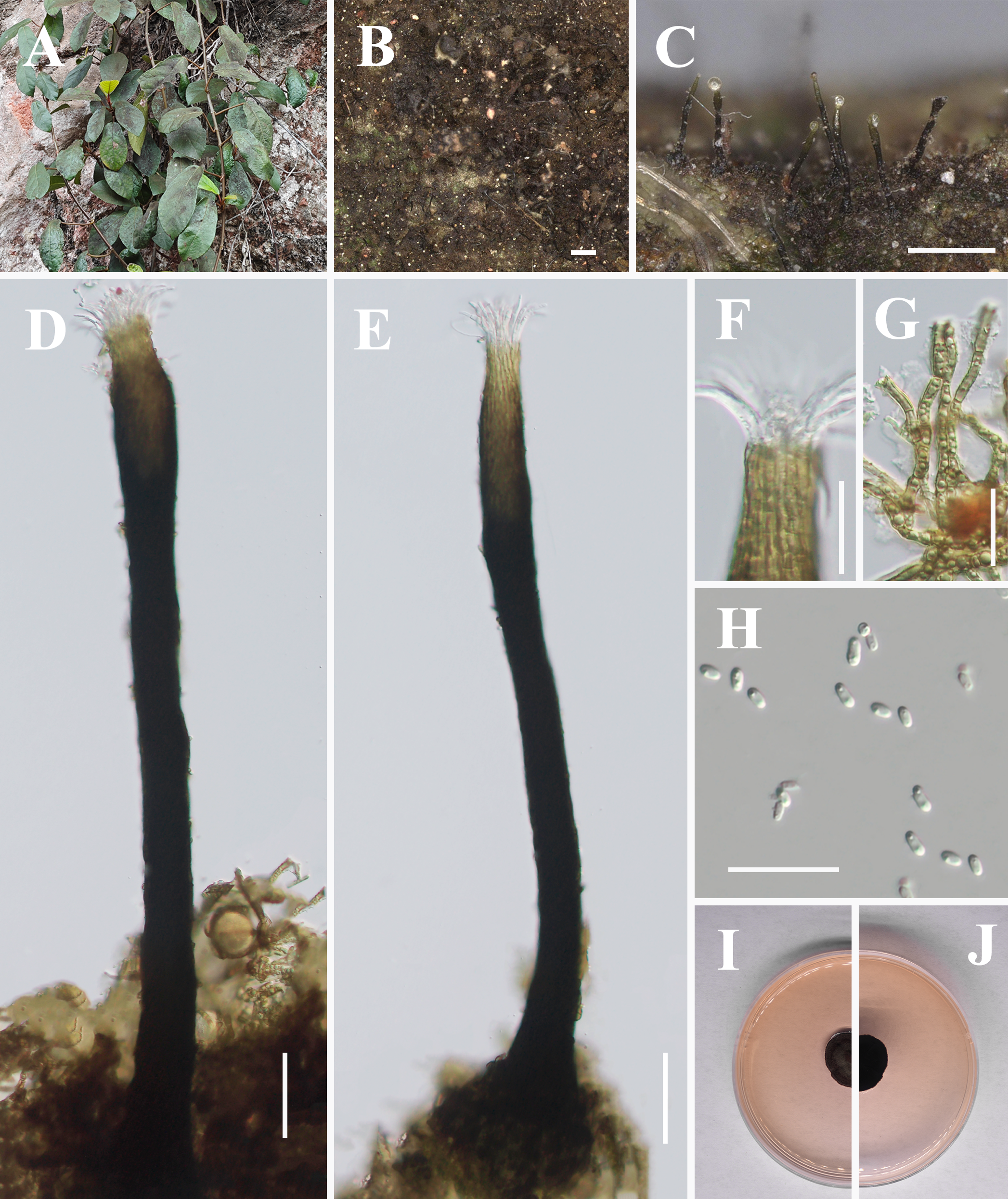

Supplement: Supplementary material 2 — Supplementary file 2 [file imafungus-17-e188051-s002.tif]

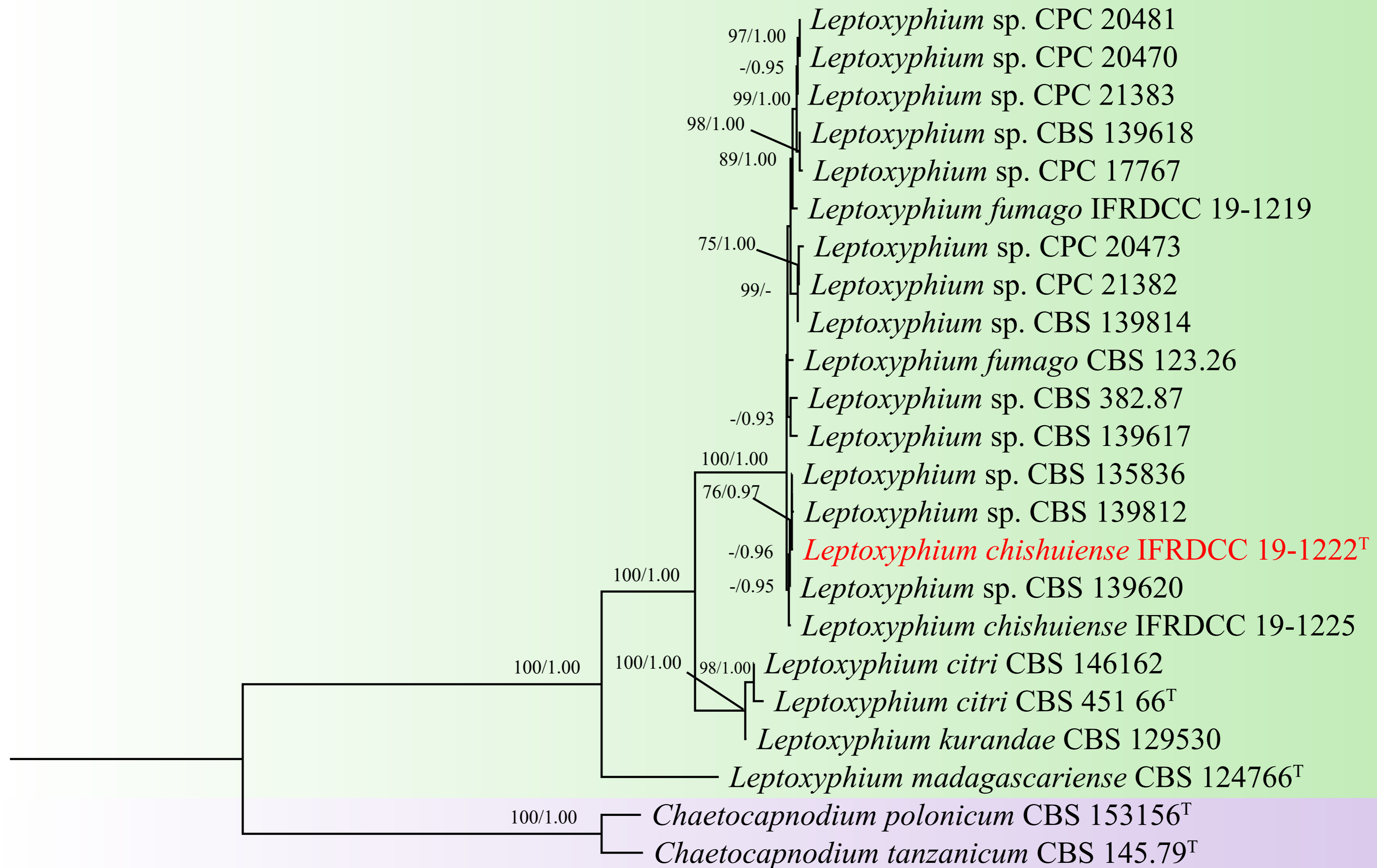

0.03

Supplement: Supplementary material 3 — Supplementary file 3 [file imafungus-17-e188051-s003.pdf]
